# Trimetazidine–Profen Hybrid Molecules: Synthesis, Chemical Characterization, and Biological Evaluation of Their Racemates

**DOI:** 10.3390/ph18091251

**Published:** 2025-08-23

**Authors:** Diyana Dimitrova, Stanimir Manolov, Iliyan Ivanov, Dimitar Bojilov, Nikol Dimova, Gabriel Marc, Smaranda Oniga, Ovidiu Oniga

**Affiliations:** 1 Department of Organic Chemistry, Faculty of Chemistry, University of Plovdiv, 24 “Tsar Assen” Str., 4000 Plovdiv, Bulgaria; dimitrovadiyana5@gmail.com (D.D.); iiiliyan@abv.bg (I.I.); bozhilov@uni-plovdiv.net (D.B.); nikol.02uzunova@gmail.com (N.D.); 2 Department of Organic Chemistry, Faculty of Pharmacy, “Iuliu Hațieganu” University of Medicine and Pharmacy, 41 Victor Babeș Street, RO-400010 Cluj-Napoca, Romania; marc.gabriel@umfcluj.ro; 3 Department of Pharmaceutical Chemistry, Faculty of Pharmacy, “Iuliu Hațieganu” University of Medicine and Pharmacy, 41 Victor Babeș Street, RO-400010 Cluj-Napoca, Romania; smaranda.oniga@umfcluj.ro; 4 Department of Therapeutic Chemistry, “Iuliu Hațieganu” University of Medicine and Pharmacy, 12 Ion Creangă Street, RO-400010 Cluj-Napoca, Romania; ooniga@umfcluj.ro

**Keywords:** trimetazidine, amide, profens, in vitro, antioxidant activity, anti-inflammatory activity, angina pectoris, HPSA, HRSA, IAD, lipophilicity, toxicity

## Abstract

**Background:** Trimetazidine is a clinically established cardioprotective agent with anti-ischemic and antioxidant properties, widely used in the management of coronary artery disease. Combining its metabolic and cytoprotective effects with the potent anti-inflammatory activity of profens presents a promising therapeutic strategy. **Methods:** Five novel trimetazidine–profen hybrid compounds were synthesized using *N*,*N*′-dicyclohexylcarbodiimide-mediated coupling and structurally characterized by NMR and high-resolution mass spectrometry. Their antioxidant activity was evaluated by hydroxyl radical scavenging assays (HRSA), and the anti-inflammatory potential was assessed via the inhibition of albumin denaturation (IAD). Lipophilicity was determined chromatographically. Molecular docking and 100 ns molecular dynamics simulations were performed to investigate the binding modes and stability in human serum albumin (HSA) binding sites. The acute toxicity of the hybrid molecules was predicted in silico using GUSAR software. **Results:** All synthesized hybrids demonstrated varying degrees of biological activity, with compound **3c** exhibiting the most potent antioxidant (HRSA IC_50_ = 71.13 µg/mL) and anti-inflammatory (IAD IC_50_ = 108.58 µg/mL) effects. Lipophilicity assays indicated moderate membrane permeability, with compounds **3c** and **3d** showing favorable profiles. Docking studies revealed stronger binding affinities of *S*-enantiomers, particularly **3c** and **3d**, to Sudlow sites II and III in HSA. Molecular dynamics simulations confirmed stable ligand–protein complexes, highlighting compound **3c** as maintaining consistent and robust interactions. The toxicity results indicate that most hybrids, particularly compounds **3b**–**3d**, exhibit a favorable safety profile compared to the parent trimetazidine. **Conclusions:** The hybrid trimetazidine–profen compounds synthesized herein, especially compound **3c**, demonstrate promising dual antioxidant and anti-inflammatory therapeutic potential. Their stable interaction with serum albumin and balanced physicochemical properties support further development as novel agents for managing ischemic heart disease and associated inflammatory conditions.

## 1. Introduction

**Figure 1:**
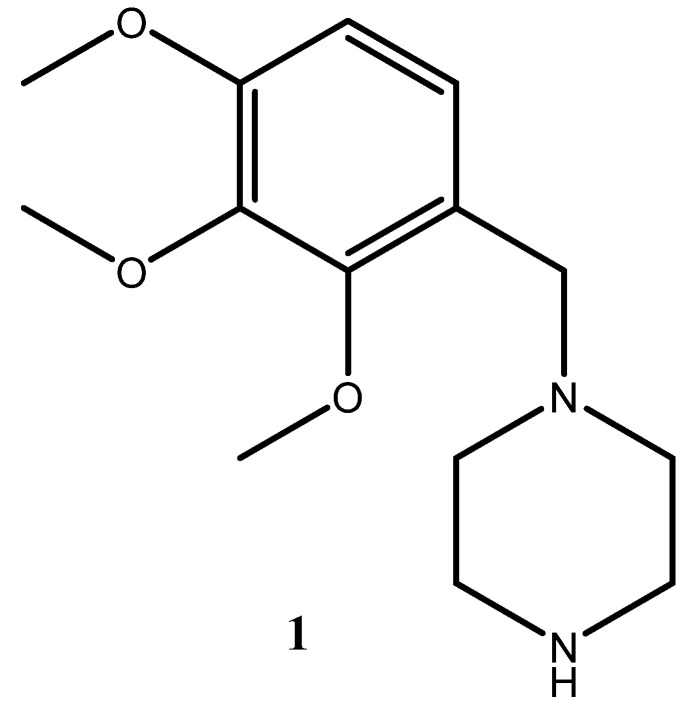
Structural formula of trimetazidine.

Trimetazidine **1** (Figure 1) is an anti-ischemic agent widely employed in the treatment of coronary artery disease due to its well-documented cardioprotective properties [1]. This compound is a piperazine-based molecule with the molecular formula C_14_H_22_N_2_O_3_ (1-[(2,3,4-trimethoxyphenyl)methyl]piperazine). Clinically, trimetazidine is marketed as dihydrochloride salt in the form of 20 mg immediate-release tablets or 35 mg modified-release tablets [2].

Across many countries, trimetazidine has been utilized in the management of angina pectoris for over four decades. Trimetazidine holds a unique place in cardiovascular therapy for heart failure due to its ability to enhance energy metabolism, setting it apart from other pharmacological agents. Numerous fundamental studies have confirmed its beneficial effects, including anti-fibrotic and anti-apoptotic actions on the myocardium, as well as notable anti-inflammatory properties [3].

**Figure 2:**
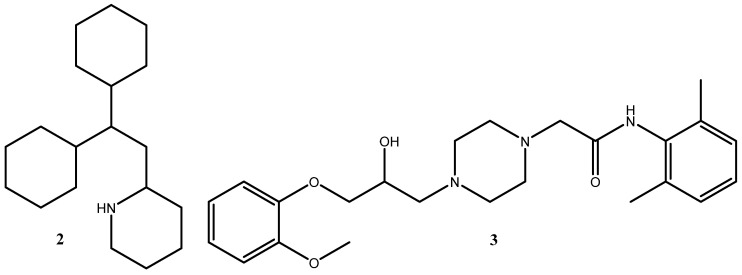
Structural formulas of pharmacological agents that inhibit fatty acid oxidation in the heart myocytes.

Trimetazidine’s mechanism of action stems from its ability to modulate cellular energy metabolism [4]. Optimization of the myocardial metabolism represents an emerging therapeutic strategy for patients with stenotic coronary artery disease. The pharmacological action of trimetazidine involves the selective inhibition of long-chain 3-ketoacyl-CoA thiolase coupled with the direct activation of pyruvate dehydrogenase. This dual mechanism effectively shifts cardiac energy metabolism from fatty acid oxidation toward glucose oxidation, thereby preserving adequate adenosine triphosphate (ATP) levels within myocardial cells, reducing intracellular acidosis, and protecting cardiomyocytes from calcium overload. Consequently, free radical-induced myocardial damage is attenuated, and the extent of myocardial necrosis is minimized [5,6,7]. Fatty acid oxidation is regulated by the plasma concentration of free fatty acids, the activity of carnitine palmitoyltransferase-1 (CPT-1), and mitochondrial β-oxidation. Pharmacological agents that inhibit cardiac fatty acid oxidation act through one of three principal mechanisms: suppression of fatty acid release from adipocytes (e.g., β-blockers), inhibition of carnitine palmitoyltransferase-1 and subsequent mitochondrial uptake of fatty acids (e.g., perhexiline **2** (Figure 2)), or direct inhibition of β-oxidation (e.g., trimetazidine **1** and ranolazine **3**) (Figure 1 and Figure 2) [8].

Studies in various animal models of atherosclerosis and diabetes have demonstrated that trimetazidine can suppress inflammatory processes. Its anti-inflammatory properties have also been documented in models of muscle atrophy and mercury-induced nephrotoxicity [9].

Furthermore, trimetazidine exerts a direct protective effect on the biological functions of endothelial progenitor cells against H_2_O_2_-induced damage. Its antioxidant and anti-inflammatory properties have also been demonstrated in the attenuation of carotid artery stenosis, achieved through the suppression of vascular smooth muscle cell proliferation and the inhibition of apoptosis in human umbilical vein endothelial cells. Additionally, trimetazidine supports endothelial function by enhancing nitric oxide production and preventing the intracellular accumulation of protons, sodium, and calcium in myocytes [10,11,12].

These findings suggest that trimetazidine may represent a suitable candidate for counteracting neuroinflammation and for preventing cognitive decline following lipopolysaccharide administration. The results open promising avenues for the development of effective adjuvant therapies for neurodegenerative dementia [13].

Multiple studies have shown that trimetazidine, whether administered alone or alongside calcium channel blockers, can significantly improve symptoms, quality of life, and ischemic indicators during stress testing in patients with chronic stable angina and ischemic cardiomyopathy when used in addition to standard therapy. Moreover, clinical trials have validated its effectiveness in refractory angina and have highlighted the added benefits of combining this metabolic agent with conventional hemodynamic treatments, including β-blockers and nitroglycerin [14].

Ischemic heart disease and heart failure are two major cardiovascular conditions with increasing prevalence, posing a significant burden on global healthcare systems [15].

In cases of heart failure with reduced ejection fraction (HFrEF), trimetazidine has been shown to improve myocardial metabolic efficiency while enhancing both systolic and diastolic cardiac function [16]. Moreover, a growing body of evidence from multiple clinical studies supports its pronounced cytoprotective properties in patients experiencing acute ischemic events [17]. Although the safety profile of trimetazidine is generally regarded as favorable, several adverse drug reactions have been documented, including gastrointestinal disturbances, nausea, vomiting, headache, hepatic dysfunction, thrombocytopenia, and agranulocytosis. These adverse effects are reported to be rare and typically reversible, with the majority not considered to be directly attributable to trimetazidine therapy. However, it has also been observed to exacerbate clinical manifestations in patients with Parkinson’s disease [18,19].

**Figure 3:**
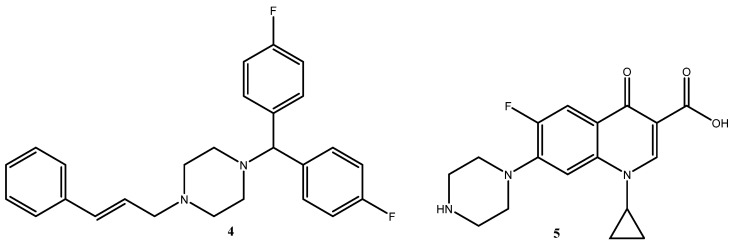
Structural formulas of flunarizine and ciprofloxacin.

The piperazine framework, as one of the simplest diazacycloalkanes, occupies an important position in the field of pharmaceutical chemistry, with substituted piperazine derivatives playing a crucial role in the development of novel therapeutics. They exhibit a broad spectrum of biological activities, including antitubercular, antibacterial, anti-inflammatory, antiviral, anticancer, antidiabetic, and antimalarial effects. Examples of drugs containing a piperazine ring include flunarizine **4**, which possesses antivasospastic properties and is broadly applied as a calcium channel blocker, and ciprofloxacin **5**, which is used to combat various bacterial strains (Figure 3) [20].

**Figure 4:**
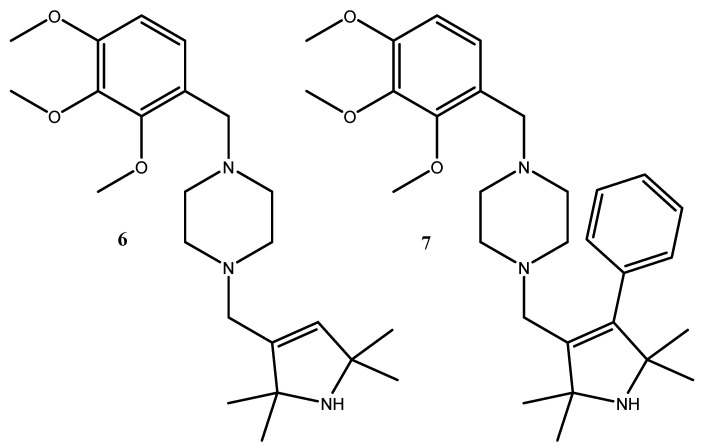
Structural formulas of trimetazidine derivatives.

Given trimetazidine’s cardioprotective and antianginal effects, the scientific literature reports the synthesis of its derivatives with the aim of investigating their potential biological properties. The authors of one study examined the efficacy of 2,2,5,5-tetramethylpyrrolinyl trimetazidine **6** and 2,2,5,5-tetramethyl-4-phenylpyrrolinyl trimetazidine **7** (Figure 4) in protecting the heart from ischemia–reperfusion-mediated injury using an isolated rat heart model. The results demonstrated that these derivatives significantly improved the recovery of ischemia–reperfusion-induced cardiac dysfunction and protected the heart from damage through their anti-ischemic and antioxidant properties [21].

**Figure 5:**
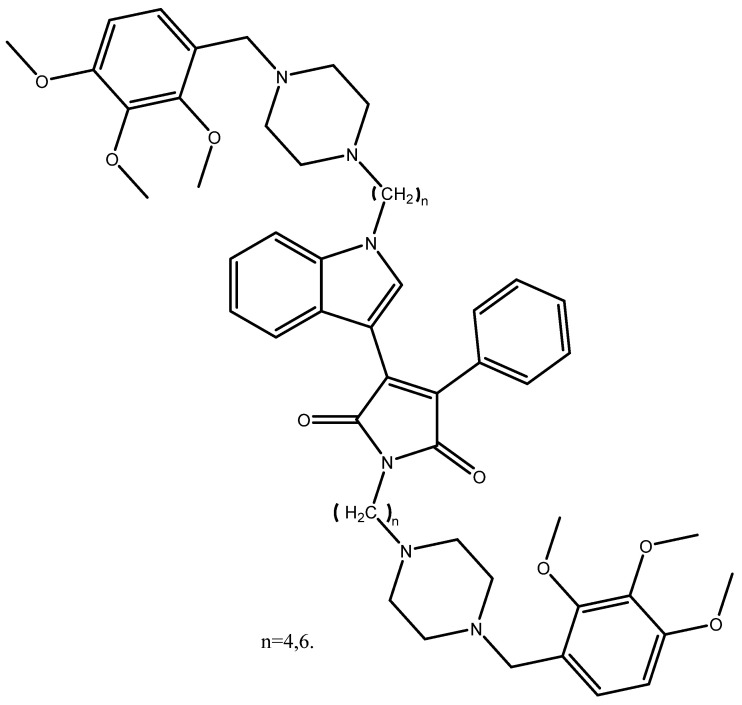
Structural formulas of maleimide derivatives with a trimetazidine fragment.

Eyilcim et al. synthesized novel maleimide derivatives containing a trimetazidine fragment (Figure 5). The anticancer potential of these compounds was subsequently evaluated in vitro using two different breast cancer cell lines [22].

Based on the above, the trimetazidine derivatives exhibit significant potential due to their enhanced cardioprotective, anti-ischemic, and antioxidant properties. These compounds not only improve myocardial recovery following ischemic injury but demonstrate promising anticancer activities, underscoring their versatility as therapeutic agents.

Inflammatory processes are increasingly recognized as underlying many different pathological conditions (e.g., heart disease, cancer, diabetes). Due to the systemic side effects of steroidal drugs, nonsteroidal anti-inflammatory drugs are often preferred for long-term inflammation treatment. They are used to manage both acute and chronic inflammation and are highly effective in most cases [23]. Profens, or 2-arylpropionic acids, are a class of nonsteroidal anti-inflammatory drugs (NSAIDs) with anti-inflammatory and analgesic activity by targeting the cyclooxygenase enzymes that mediate inflammatory responses. These profens constitute a group of analgesic, antipyretic, and anti-inflammatory agents widely used in both humans and animals, as they do not induce sedation, respiratory depression, or dependence. This group includes ibuprofen, ketoprofen, naproxen, and flurbiprofen, among others [24]. They are formulated as a racemic mixture of R- and S-enantiomers; however, the analgesic effect is primarily associated with one enantiomer, while the other is largely responsible for side effects or toxicity. From a medical perspective, the enantiomer with the desired biological activity should be administered to avoid unnecessary drug intake and to minimize adverse effects [25]. Due to the presence of R- and S-forms of profens, a phenomenon known as the metabolic chiral inversion of these drugs can occur in the human body. This takes place in the presence of enzymes or metabolites that cause a shift in the enantiomeric composition of the chiral drug [26]. Although often used as racemic mixtures, the S-enantiomers of profens are the primary bioactive enantiomers responsible for cyclooxygenase inhibition, whereas the R-enantiomers are generally not considered cyclooxygenase inhibitors [27].

Considering the presented evidence, the synthesis of novel hybrid compounds combining trimetazidine and profens represents an interesting direction in medicinal chemistry. Trimetazidine’s well-documented cardioprotective, anti-ischemic, and metabolic-modulating properties, together with the potent anti-inflammatory and analgesic effects of profens, offer a unique therapeutic potential. By merging these pharmacophores into hybrid molecules, it may be possible to achieve dual cardioprotective and anti-inflammatory actions, to enhance efficacy, and to reduce the drawbacks associated with racemic profen formulations, including adverse effects and chiral inversion. Such hybrids could open promising avenues for the development of next-generation agents for the management of ischemic heart disease, chronic inflammation, and other related conditions.

## 2. Results and Discussion

### 2.1. Synthesis

**Figure 6:**
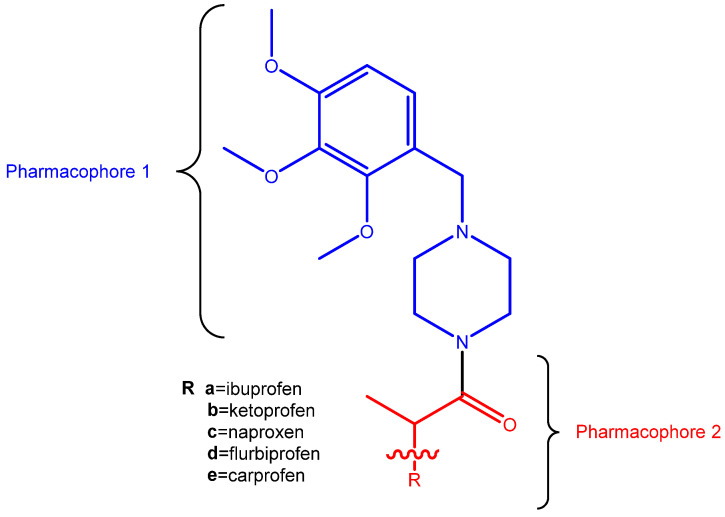
General structural formula of the target hybrid molecules.

Given the distinct yet complementary mechanisms of action of trimetazidine and profens, there is growing interest in the design of hybrid molecules that incorporate structural elements from both classes. In this context, the primary objective of the present study was formulated to synthesize novel hybrid molecules combining the two pharmacophores—Pharmacophore 1 (trimetazidine) and Pharmacophore 2 (profens) (Figure 6). Such molecules are expected to simultaneously target multiple pathological processes, and may hold therapeutic potential for conditions such as cardiovascular diseases with inflammatory components, neurodegenerative disorders, or certain types of chronic inflammatory states.

By integrating the anti-ischemic and antioxidant properties of trimetazidine with the anti-inflammatory activity of profens, the hybrid molecule has the potential to deliver enhanced therapeutic efficacy compared to monotherapies.

This work investigates the synthesis of five novel amides through a simple and efficient approach employing *N*,*N*′-dicyclohexylcarbodiimide (DCC) as a coupling reagent. DCC is a widely utilized reagent in organic synthesis, valued particularly for its ability to activate carboxylic acids, thereby rendering them more susceptible to nucleophilic attack by amines and facilitating the formation of amide bonds. During this process, *N*,*N*′-dicyclohexylurea is generated as a byproduct, which typically precipitates from the reaction mixture and can be readily removed via filtration. A key advantage of employing DCC lies in its high efficiency under mild reaction conditions.

**Scheme 1:**
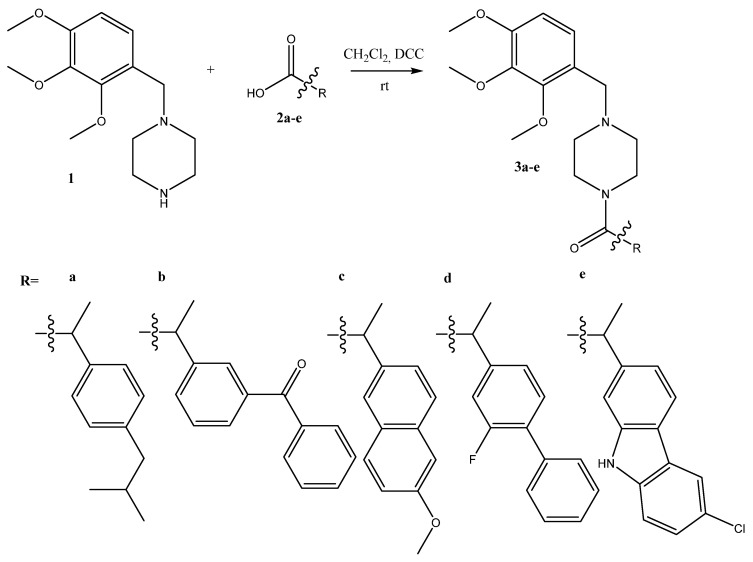
Synthesis of compounds **3a**–**e** via *N*,*N*′-dicyclohexylcarbodiimide-mediated coupling.

In the present study, DCC reacts with the carboxylic group of the respective profen, leading to the formation of an activated acylating intermediate, which subsequently undergoes nucleophilic substitution with the secondary amine group of trimetazidine, ultimately yielding the desired amide bond (Scheme 1). All profens utilized in the synthesis were racemic and not resolved into individual enantiomers.

Dichloromethane (DCM) was selected as the solvent of choice for conducting the reactions. The target amides were isolated in high yields ranging from 82% to 98%, and their purity was confirmed, with structural elucidation accomplished by ^1^H and ^13^C NMR spectroscopy (Bruker, Billerica, MA, USA) (Figures S1–S10), as well as mass spectrometry (MS) (Bremen, Germany) (Figures S11–S15).

### 2.2. In Vitro Biological Evaluation

The biological activity of the synthesized compounds was evaluated using three complementary assays: hydrogen peroxide scavenging activity (HPSA), hydroxyl radical scavenging activity (HRSA), and the inhibition of albumin denaturation (IAD). IC_50_ values (µg/mL) were employed as quantitative indicators of activity. A statistical analysis was performed by one-way ANOVA followed by Duncan’s post hoc test (α = 0.05) to identify homogeneous subgroups and to determine statistically significant differences among the samples.

#### 2.2.1. Hydrogen Peroxide Scavenging Activity (HPSA)

Reactive oxygen species (ROS), which include oxygen-centered radicals, non-radical oxidants, and singlet oxygen (^1^O_2_), are inevitable byproducts of oxidative metabolism in all living organisms [28]. ROS are highly reactive and can quickly interact with various biological molecules, such as proteins, lipids, lipoproteins, and nucleic acids. Elevated levels of ROS can cause oxidative stress, leading to the cellular and tissue damage implicated in aging, atherosclerosis, cancer development, and mutagenesis [29]. Fortunately, the prominent antioxidant enzymes of the endogenous ROS defense system can effectively mitigate the harmful effects of free radical attacks; however, they are unable to completely prevent oxidative damage. Therefore, exogenous antioxidants play a vital role in maintaining overall health [30]. However, the application of synthetic antioxidants has been progressively limited due to potential health risks, including protein and DNA damage, among other toxic effects [31]. In addition to oxidative stress mechanisms, the inflammatory process both promotes and amplifies the production of reactive oxygen species (ROS). A key factor in this process is the generation of superoxide anion radicals, which subsequently lead to the formation of other ROS, such as hydrogen peroxide (H_2_O_2_) [32].

**Figure 7:**
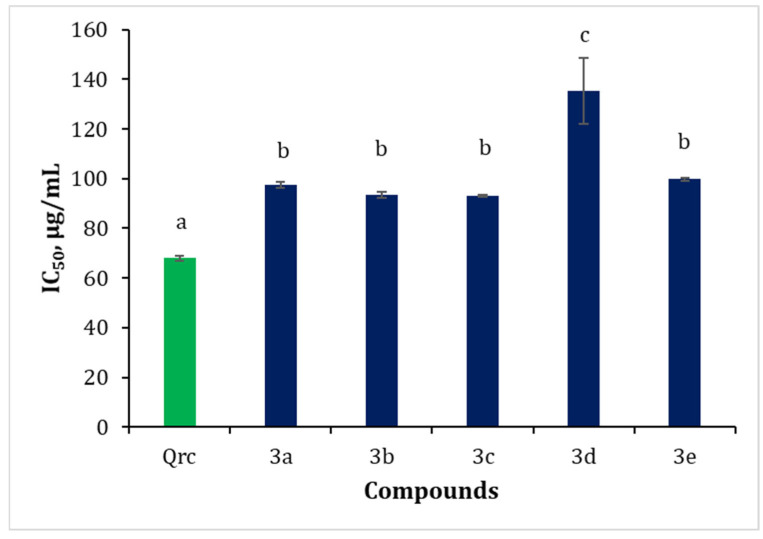
HPSA results of the trimetazidine hybrids. The values are presented as IC_50_, µg/mL. Quercetin (Qrc) was used as the standard. Different letters for the same method indicate significant difference at *p* < 0.05 levels by Duncan’s test. Duncan’s test compares the average values of the groups and classifies them by significance levels. Group average values that do not differ significantly are united into homogeneous subgroups.

The present study assessed the potential of trimetazidine derivatives to inhibit the harmful effects of reactive oxygen species (ROS), with a particular focus on hydrogen peroxide (H_2_O_2_). Quercetin, well known for its antioxidant properties, was employed as a reference standard. The results of the hydrogen peroxide scavenging activity (HPSA) assay are presented in Figure 7 and Table S1. The IC_50_ values of the synthesized trimetazidine derivatives ranged from 93.07 to 135.25 µg/mL. In comparison with quercetin, the trimetazidine derivatives demonstrated relatively low antioxidant activity. The statistical evaluation of the antioxidant activity of the trimetazidine derivatives was performed using Duncan’s test. The HPSA results revealed statistically significant differences among the tested samples (ANOVA: F = 46.64, *p* < 0.001). Duncan’s post hoc analysis grouped the samples into three homogeneous subgroups. The control sample, quercetin (68.27 µg/mL), was the only compound classified in the first group, indicating that it was statistically the most active. The synthesized trimetazidine derivative compounds **3a**, **3b**, **3c**, and **3e** formed the second subgroup, with IC_50_ values ranging from 93.07 to 99.75 µg/mL, showing no statistically significant differences among them. Compound **3d** (135.24 µg/mL) was classified independently in the third subgroup, underscoring its markedly lower antioxidant activity (Figure 7). These findings demonstrate that only quercetin exhibits a strong capacity to neutralize hydrogen peroxide, most likely attributable to the presence of multiple phenolic hydroxyl groups, which facilitate electron donation and stabilization of the resulting radical. By contrast, the trimetazidine derivatives display moderate to low activity, likely due to more limited electron delocalization or reduced polarity.

#### 2.2.2. Hydroxyl Radical Scavenging Activity (HRSA)

**Figure 8:**
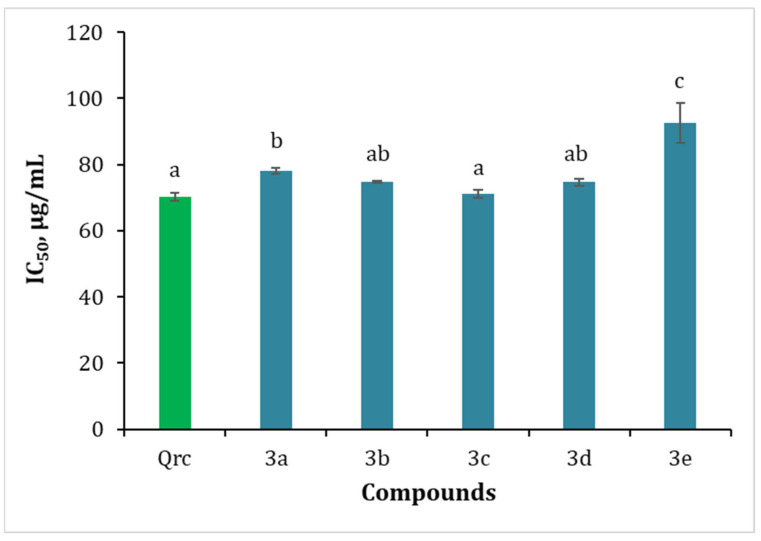
Results of the HRSA of trimetazidine derivatives. The values are presented as IC_50_, µg/mL. Quercetin (Qrc) was utilized as the standard. Different letters for the same method indicate significant difference at *p* < 0.05 levels by Duncan’s test. Duncan’s test compares the average values of the groups and classifies them by significance levels. Group average values that do not differ significantly are united into homogeneous subgroups.

The hydroxyl radical (^•^OH) represents the most potent oxidizing species generated within the spectrum of reactive oxygen species (ROS). It arises through one-electron reductions of molecular oxygen (O_2_) during cellular metabolic processes, and constitutes a principal mediator of cytotoxicity in aerobic organisms, including humans [33]. The highly reactive ^•^OH radicals induce protein carbonylation, damage to DNA structure, and disruption of the cell membrane. They are also responsible for the oxidation of essential polyunsaturated fatty acids, a process commonly referred to as lipid peroxidation. Figure 8 illustrates a pronounced difference in antioxidant activity among the compounds under investigation (Figure 8, Table S1).

The HRSA assay likewise revealed statistically significant differences among the tested compounds (ANOVA: F = 30.11, *p* < 0.001). An analysis of the HRSA data using Duncan’s test identified four homogeneous subgroups. Quercetin (70.11 µg/mL) and compound **3c** (71.13 µg/mL) exhibited the lowest IC_50_ values and were classified in the first subgroup, indicating that their antioxidant capacity was statistically the highest. They were followed by compounds **3b** and **3d**, forming the second subgroup, with IC_50_ values of approximately 74.6–74.7 µg/mL. Compound **3a** (77.98 µg/mL) was placed in a separate third subgroup, while compound **3e** (92.56 µg/mL) stood alone in the fourth subgroup, demonstrating the weakest activity.

The HRSA results suggest that quercetin and compound **3c** possess comparable efficiency in scavenging hydroxyl radicals, likely due to the presence of electron-rich aromatic systems capable of neutralizing the highly reactive ^•^OH radicals. By contrast, compound **3e**, with its higher IC_50_ value, may lack suitable electron-donating groups or exhibit structural constraints that hinder effective interaction with the radicals.

#### 2.2.3. Inhibition of Albumin Denaturation (IAD)

The anti-inflammatory activity can be evaluated using several biochemical methods, including the inhibition of albumin denaturation (IAD) assay. The IAD method enables the assessment of a compound’s capacity to suppress inflammatory processes by inhibiting the specific enzymes or signaling pathways associated with inflammation. Such an evaluation is essential for identifying potential therapeutic agents with anti-inflammatory properties and for advancing the development of treatments for chronic inflammatory disorders.

**Figure 9:**
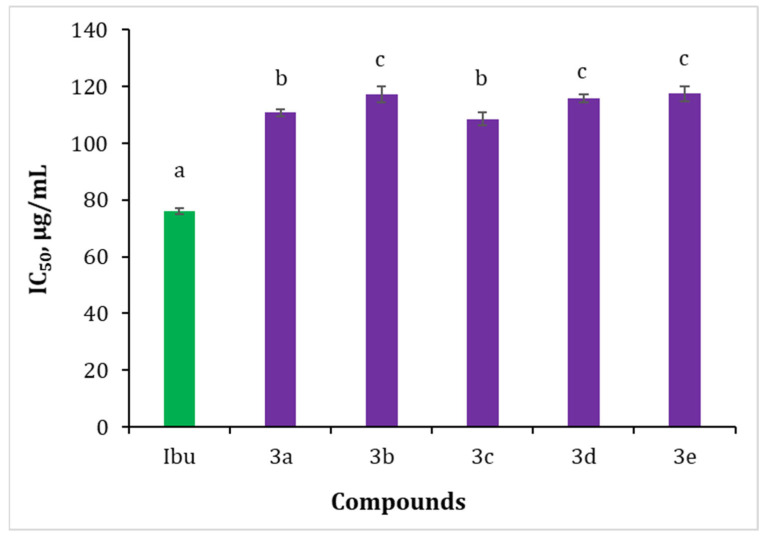
IAD results of the trimetazidine hybrids. Values are presented as IC_50_, µg/mL. Ibuprofen (Ibu) was used as the standard. Different letters for the same method indicate significant difference at *p* < 0.05 levels by Duncan’s test. Duncan’s test compares the average values of the groups and classifies them by significance levels. Group average values that do not differ significantly are united into homogeneous subgroups.

The results for the synthesized trimetazidine derivatives are presented in Figure 9. The IAD data are expressed as IC_50_ values. Ibuprofen, a well-established anti-inflammatory agent, was employed as a reference standard for a comparison with the newly synthesized trimetazidine derivative compounds **3a**–**e**. The IC_50_ value of ibuprofen, determined via the IAD assay, was calculated as 76.05 µg/mL (Figure 9, Table S1). The analysis revealed that the IC_50_ values of the trimetazidine derivatives varied between 108.58 and 117.51 µg/mL (Figure 9, Table S1).

The post hoc analysis of the in vitro anti-inflammatory activity assessed via the IAD method revealed significant differentiation among the trimetazidine derivatives (ANOVA: F = 176.58, *p* < 0.001). Ibuprofen was classified in the first homogeneous group, demonstrating the highest activity. Trimetazidine derivative compounds **3a** (110.85 µg/mL) and **3c** (108.58 µg/mL) formed the second group, exhibiting intermediate activity, whereas compounds **3b**, **3d**, and **3e** (IC_50_ ~115–117 µg/mL) were placed in the third group, reflecting the weakest ability to inhibit albumin denaturation. Ibuprofen showed a marked superiority over the tested derivatives in inhibiting protein denaturation—an indirect marker of inflammation. Nevertheless, the new derivatives with lower IC_50_ values (compounds **3a** and **3c**) remain potentially valuable, particularly if they demonstrate synergistic effects or improved bioavailability.

The post hoc analysis using Duncan’s test distinctly differentiated the efficacy of the compounds across each assay. The series of compounds **3a–e** exhibited varying degrees of biological activity depending on the applied methodologies. Although none surpassed the reference standards, compound **3c** demonstrated antioxidant activity against hydroxyl radicals that was statistically comparable to that of the reference compound, alongside moderate activity in the other assays. These results position compound **3c** as the most promising candidate for further development.

#### 2.2.4. Lipophilicity

Lipophilicity, commonly expressed as LogP, denotes the equilibrium ratio of a compound’s concentration between two immiscible phases, typically an oil (organic) phase and an aqueous (liquid) phase [34]. Lipophilicity constitutes a fundamental physicochemical parameter in drug development, profoundly impacting the absorption, distribution, metabolism, excretion, and toxicity (ADMET) characteristics of pharmaceutical agents. Within the drug discovery process, the optimization of lipophilicity is critical to achieving favorable pharmacokinetic and pharmacodynamic profiles, thereby enhancing the therapeutic potential and safety of candidate compounds [35]. There has been a growing emphasis on designing drug candidates with higher lipophilicity to achieve the desired levels of selectivity and potency. This trend primarily stems from the inherently lipid-rich environment of many biological targets, which often favors the interaction of lipophilic compounds and enhances their binding affinity [36].

In this study, the lipophilicity of the synthesized trimetazidine–profen derivatives was evaluated using a practical and widely applied technique—reversed-phase thin-layer chromatography (RP-TLC)—following the methodology outlined by Pontiki and Hadjipavlou-Litina [37].

The experimental results are summarized in Table S1. The lipophilicity of the compounds, as determined by their RM values, increased in the following order: **3d** (1.45) < **3b** (1.55) = **3e** (1.55) < **3c** (1.61) < **3a** (1.68). Among the tested compounds, **3a** exhibited the highest lipophilicity, followed by **3c**, whereas **3d** was the least lipophilic. This trend highlights a notable variability in lipophilic character, which is likely attributable to structural and functional group differences among the compounds. Notably, compounds with elevated RM values tend to display enhanced interactions with lipid membranes [38].

#### 2.2.5. Molecular Docking

The affinity of both the R- and S-enantiomers of compounds **3a**–**e** to the albumin binding sites, resulted from the molecular docking, as determined using AutoDock Vina v 1.1.2, are summarized in Table 1, while the corresponding results produced using AutoDock v 4.2 are presented in Table 2, showing the top-ranked binding conformation from the most populated cluster for each compound, ensuring result reproducibility.

Analyzing the overall binding affinity of the compounds to the four studied sites by both software used, it can be seen that the compounds have the best affinity to Sudlow sites II and III, an intermediate affinity for the cleft site, and the lowest affinity for the Sudlow site I. No clear overall difference in terms of affinity difference to the binding sites can be identified between the type of enantiomer of the compounds, but on the cleft site and on Sudlow site II, the S-enantiomers have better binding affinity to the two sites, when compared to the R-enantiomers. Regarding the low affinity of the compounds for the Sudlow site I, as a result of the clustering analysis of the binding poses from the molecular docking performed with AutoDock, it stands out as the low number of other conformations in the same cluster with the top binding pose, indicating a high dispersion of the predicted poses. The high dispersion of the binding poses of the compounds in Sudlow site I was supplementarily confirmed by the high number of other clusters (>18 other clusters—data not presented). Overall, the binding affinities found using AutoDock Vina and AutoDock follow a similar trend, although they use different search protocols.

**Figure 10:**
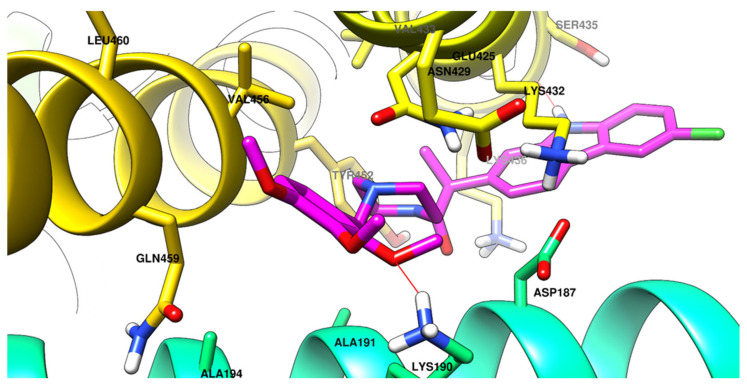
The top ranked conformation of compound **3e[S]** (carbon atoms depicted in magenta) in the cleft site, where it had the strongest affinity among the current series of compounds. The compound was accommodated in an environment with numerous and complex polar interactions between the amino acids, for example, the salt bridges between the sidechains of Asp187, Glu425, and Lys432. In the same region other polar sidechains are present—Asp187, Lys436, Asn429, or Tyr452, which can be considered less favorable for stable interactions with compound **3e[R]**. Some of the polar amino acids in the respective site are interacting with the ligand—the positively charged sidechain of Lys190 was predicted to interact with one of the oxygen atoms from the ether groups from the trimethoxy moiety of the molecule, while the hydrogen from the amine of the carbazole moiety is predicted to make a hydrogen bond with the peptide bond Ser436–Lys436.

**Figure 11:**
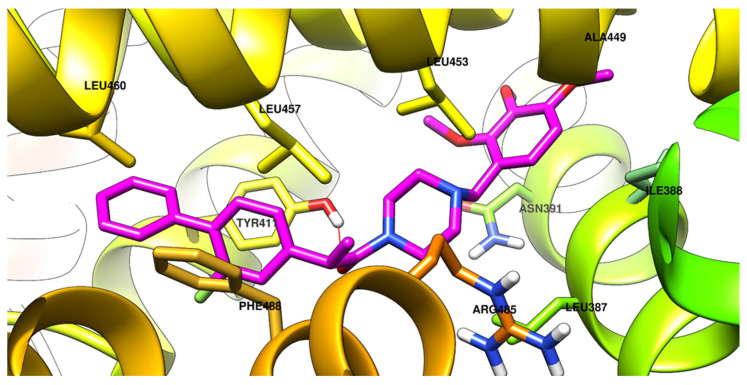
The top ranked conformation of compound **3d[S]** (carbon atoms depicted in magenta) bound in Sudlow site II of albumin, where it had the strongest affinity among the current series of compounds. The present compound is predicted to be involved in a hydrogen bond with Tyr411 as the acceptor via the amide oxygen atom. The molecule is found in a hydrophobic environment, surrounded by several sidechains of hydrophobic amino acids, such as Phe488, Leu387, Ile388, Ala449, Leu453, Leu457, and Leu460.

**Figure 12:**
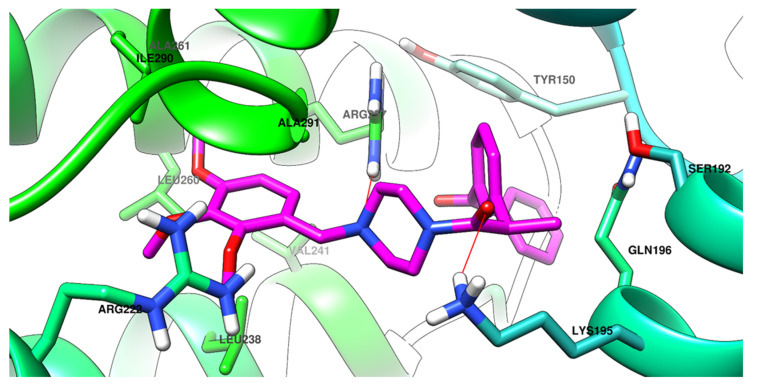
The top ranked conformation of compound **3b[R]** (carbon atoms depicted in magenta) bound in Sudlow site I of albumin, where it had the strongest affinity among the current series of compounds. Two polar contacts were predicted to appear—one between amide oxygen and the positively charged sidechain of Lys195 and the second between the positively charged sidechain of Arg257 and one of piperazine nitrogen atoms. The trimethoxyphenyl region of the ligand seems to accommodate well in a mainly hydrophobic region, surrounded by Leu238, Val241, Ala291, Leu260, and Ile290.

**Figure 13:**
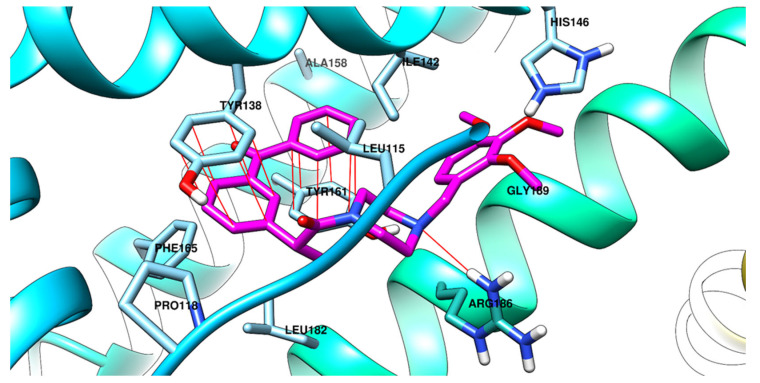
The top ranked conformation of compound **3b[R]** (carbon atoms depicted in magenta), bound in site 3 of albumin, where it had the strongest affinity among the current series of compounds. The benzenes from the diphenyl-ketone moiety of the compound are predicted to be involved in π–π stacking with sidechains of Tyr138 and Tyr161, respectively. The positively charged sidechain of Arg186 is predicted to be involved in one polar contact with a nitrogen atom from piperazine.

For each binding site analyzed, the top predicted binding pose of the compound with the highest binding affinity for the respective site is shown in Figure 10, Figure 11, Figure 12 and Figure 13.

#### 2.2.6. Molecular Dynamics

The stability of the albumin–ligand complexes, each incorporating the top-ranked binding conformation at the four investigated sites, was assessed through molecular dynamics simulations. System stability during the simulations was evaluated by monitoring several parameters: the average root mean square deviation (RMSD) of the ligand’s heavy atoms, the RMSD of the protein backbone, the protein’s atoms radius of gyration (Rg), and the number of hydrogen bonds formed between the ligands and albumin throughout the trajectory. To preliminary evaluate the stability of the ligand–protein complexes, the RMDS of the heavy atoms of the ligand was assessed with a 0.5 nm arbitrary threshold. The complexes of **3c[R]**, **3c[S]**, and **3e[R]** docked into site 3 exhibited high movement of the ligand in the respective site; therefore, for the respective ligands, the molecular dynamics study was repeated, but with the same compound bound in the second-ranked site—Sudlow site I for all compounds. It should be mentioned that the compounds have a high degree of flexibility, due to the relative flexible aliphatic piperazine central ring, which has flexible substituents with sp^3^ carbon atoms on both piperazine nitrogen atoms.

The numerical analysis of the molecular dynamics is presented as follows: Table 3 summarizes the RMSD values of the heavy atoms of the ligands bound to HSA, while Table 4 centralizes the RMSD values of the atoms from the protein backbone in both the apo form and when complexed with the ligands. Table 5 outlines the protein’s radius of gyration in its apo state and in complexes with the ligands, and Table 6 presents the average number of hydrogen bonds formed between albumin and the respective ligands. The graphical depiction of the respective four types of data centralized in the respective tables are presented in detail in the supplementary materials (Figures S16–S25).

For each binding site, the chimeric complex between the top binding conformation to the respective site was constructed and the stability was evaluated. Because of the large RMSD of the ligands **3c[R]**, **3c[S]**, and **3e[R]** in the binding site 3 (higher than 0.5 nm), the second ranking conformation was taken, but in another site (Sudlow site II) which exhibited much better stability during the simulation. From the total 13 molecular simulations which were performed, 10 of them exhibited good overall stability of the ligand in the targeted sites of HSA, while the other 3 exhibited unacceptable movement of the ligand and were discarded for further analysis. For the respective three molecular dynamics simulations, only the RMSD of the ligand was presented, and no further analysis was presented.

The RMSD of the heavy atoms of the ligands was lower in Sudlow site II when compared to the site III. The lowest RMSD was identified for **3d[S]** in Sudlow site II (0.23 nm), while the highest RMSD was identified for **3a[R]** in the 3 site (0.49 nm). A detailed analysis of the trajectory of **3a[R]** during the simulation indicated an initial repositioning in the site in the first 30 ns of simulation. After that, the RMSD of the ligand had some fluctuations, but lower, with a 0.42 nm on average in the 30–100 ns timeframe. Taking into account the flexibility of the compounds in the present series, it can be considered within the acceptable maximal limits. An initial repositioning of the ligand was identified for the ligands **3b[S]** (Figure S19) and **3e[R]** (Figure S24) in the first 20 ns of the simulation, after that the ligand exhibited the acceptable movement to be considered as a stable bond.

The stabilization effect of the HSA backbone was identified in the RMSD of the backbone of the protein was evaluated for compounds **3b-e**, being lower than for the apo. Interestingly, the RMSD of the backbone of HSA was higher for both stereoisomers of compound **3a**, the smallest compound when compared to compounds **3b-e**, which are bulkier. On the other hand, when analyzing the radius of gyration of the amino acids of the protein, there was no significant increase for the compound **3a R** and **S** complexes, when compared to the apo form. Overall, the radii of gyration calculated for all complexes are scattered near the apo value (2.78 nm), with the lowest value 2.72 nm for the complex with **3c[R]**.

In terms of hydrogen bonding, compound **3b[R]** stood out with the highest hydrogen bonding with HSA (on average 2.27 hydrogen bonds). While, for compound **3a** and **3c**, the average hydrogen bonds to protein is somehow equilibrated between the two enantiomers (0.91 bonds for **3a[R]**, 0.66 bonds for **3a[S]**, 0.29 bonds for **3c[R]**, and 0.37 bonds for **3c[S]**), but for the compounds **3d** and **3e**, which are less flexible, an impressive difference was identified between the two enantiomers—no hydrogen bonds were identified for both R-enantiomers, but 0.61 on average bonds for compound **3d[S]** and 0.98 on average bonds for compound **3e[S]** were identified.

#### 2.2.7. Acute Rat Toxicity Prediction Using GUSAR Software

Trimetazidine is a widely used anti-ischemic drug known for its favorable safety profile. However, recent studies have highlighted potential toxicity concerns, particularly regarding its interactions with human serum albumin (HSA). A 2023 study by Barseem et al. [39] utilized fluorescence spectroscopy and molecular docking to investigate trimetazidine’s binding affinity to HSA, revealing that it preferentially binds to subdomain III A (site II) of HSA. This interaction could influence the drug’s pharmacodynamics and pharmacokinetics, potentially affecting its therapeutic efficacy and safety [39]. Regarding clinical safety, trimetazidine is typically well-tolerated. However, rare adverse effects have been reported, including gastrointestinal disturbances, dizziness, and, in isolated cases, neurological symptoms, such as tremors and parkinsonism. These side effects are usually reversible upon discontinuation of the drug [19,40,41]

Profens, such as ibuprofen, ketoprofen, naproxen, flurbiprofen, and carprofen, are widely used NSAIDs with analgesic and anti-inflammatory properties. Despite their efficacy, they carry potential toxicity risks, particularly with overdose or prolonged use. Common adverse effects include gastrointestinal disturbances, such as nausea, vomiting, abdominal pain, and ulceration, due to inhibition of cyclooxygenase enzymes and reduced protective prostaglandins [42]. Renal toxicity may occur from impaired prostaglandin-mediated renal blood flow, while hepatic reactions, particularly with carprofen, are rare but can include acute hepatic necrosis [43]. Central nervous system effects, like dizziness and confusion, are generally reversible. In veterinary medicine, NSAID toxicity is notable; carprofen can cause gastrointestinal and hepatic effects in dogs, while ibuprofen and naproxen are highly toxic to cats and dogs [44].

Given the potential toxicity concerns associated with both trimetazidine and profens, including rare gastrointestinal, hepatic, renal, and central nervous system effects, we evaluated the acute toxicity of the newly synthesized trimetazidine–profen hybrid molecules. Acute rat toxicity was predicted using GUSAR software 2009 (Plovdiv, Bulgaria) to assess their safety profile, taking into account the known pharmacological actions and reported adverse effects of the parent compounds [19,39,40,41,42,43,44].

GUSAR software was developed to generate QSAR/QSPR models using appropriate training datasets. It enables an in silico prediction of LD_50_ values in rats for four routes of administration: oral, intravenous, intraperitoneal, and subcutaneous [45].

**Figure 14:**
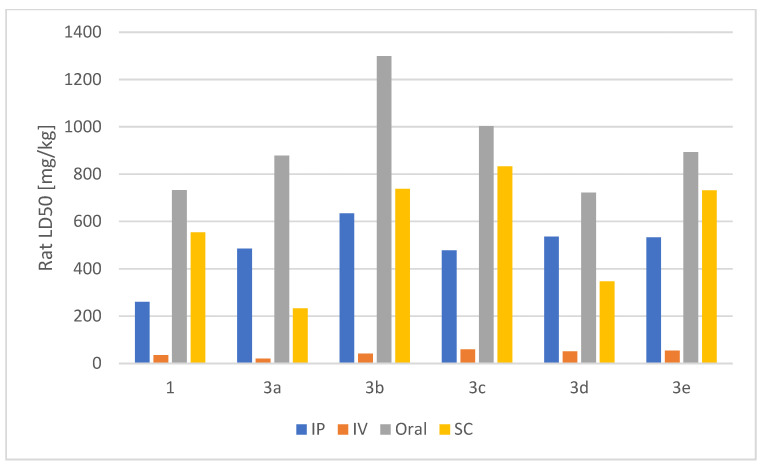
Acute rat toxicity estimated using GUSAR.

We employed the software to perform quantitative in silico predictions of the LD_50_ values for the synthesized trimetazidine–profen hybrids **3a**–**e**. The results, expressed as LD_50_ values in mg/kg, are summarized in Table 7 and Figure 14.

Based on the in silico GUSAR predictions, the synthesized trimetazidine–profen hybrid compounds **3a**–**e** generally exhibit equal or lower acute toxicity compared to the parent trimetazidine **1**. Intraperitoneal LD_50_ values for all hybrids are higher than that of trimetazidine, indicating reduced toxicity. For intravenous administration, most hybrid compounds (**3b–e**) show decreased toxicity relative to trimetazidine, whereas compound **3a** displays a lower LD_50_, suggesting greater toxicity by this route. Orally, all hybrids are less toxic, with compounds **3b**–**d** showing an increase in LD_50_, placing them in the OECD category of practically non-toxic, while compounds **3a** and **3e** show modest improvement. Subcutaneous predictions are more variable: compounds **3b**, **3c**, and **3e** are less toxic, compound **3d** is slightly more toxic, and compound **3a** is markedly more toxic, though the latter’s value lies outside the model’s applicability domain. Overall, these results suggest that the new derivatives, particularly compounds **3b**–**d**, have a favorable acute toxicity profile—especially for oral administration—although certain route-specific liabilities, especially for compounds **3a** (IV and SC) and **3d** (SC), warrant caution and further experimental validation.

Using a single composite metric across routes (geometric mean of the four LD_50_ values; lower = more toxic), the decreasing toxicity order is: **3a** > **1** > **3d** > **3e** ≈ **3c** > **3b** (approx. geometric-mean LD_50_s in mg/kg: **3a** = 212; **1** = 246; **3d** = 287; **3e** = 369; **3c** = 391; **3b** = 398); note that two inputs are out-of-domain for the model (**3a**, SC; **3e**, IP), so the relative placement of compounds **3e** vs **3c** is effectively a tie within prediction uncertainty.

## 3. Materials and Methods

### 3.1. General

The reagents were purchased from commercial suppliers (Sigma-Aldrich S.A., Sofia, Bulgaria and Riedel-de Haën, Sofia, Bulgaria) and used as received without further purification. NMR spectra were recorded on a Bruker NEO 400 spectrometer (400/100 MHz for ^1^H/^13^C; BAS-IOCCP, Sofia; Bruker, Billerica, MA, USA). All compounds were analyzed in DMSO-*d*_6_ at 400 MHz for ^1^H-NMR and 101 MHz for ^13^C-NMR. Chemical shifts (δ) are reported in parts per million (ppm) relative to tetramethylsilane (TMS, δ = 0.00 ppm) as an internal standard, and coupling constants (*J*) are expressed in hertz (Hz). All spectra were acquired at room temperature (295 K). High-resolution mass spectrometry (HRMS) analyses were performed on a Q Exactive™ Plus mass spectrometer equipped with a heated electrospray ionization source (HESI-II) (Thermo Fisher Scientific, Bremen, Germany), coupled to a Dionex UltiMate 3000 RSLC ultrahigh-performance liquid chromatography (UHPLC) system (Thermo Fisher Scientific, Inc., Waltham, MA, USA). The UHPLC system comprised a 6-channel degasser (SRD-3600), high-pressure gradient pump (HPG-3400RS), autosampler (WPS-3000TRS), column compartment (TCC-3000RS), and a narrow-bore Hypersil GOLD™ C18 column (2.1 × 50 mm, 1.9 µm). Thin-layer chromatography (TLC) analyses were performed using precoated Fluka silica gel 60 plates (0.2 mm, Merck KGaA, Darmstadt, Germany).

Water for the HPLC was obtained using a Millipore purifier (Millipore, Burlington, MA, USA). Potassium dihydrogen phosphate, phosphoric acid, dipotassium hydrogen phosphate, ibuprofen, potassium chloride, quercetin, sodium chloride, hydrogen peroxide, ferrous sulfate, phenanthroline, Tween 80, and DMSO were purchased from Sigma-Aldrich, Taufkirchen, Germany. Human albumin 20%—BB, 200 g/L was supplied from BB-NCIPD Ltd., Sofia, Bulgaria.

### 3.2. Synthesis

Into a dichloromethane solution containing the corresponding profen (1 mmol), *N*,*N*′-dicyclohexylcarbodiimide (1 mmol, 0.206 g) was introduced, and the mixture was stirred for 10 min using a magnetic stirrer. Subsequently, trimetazidine (1 mmol, 0.266 g) was introduced, and the reaction mixture was further stirred for 50 min. The reaction progress was monitored by thin-layer chromatography (TLC). Upon completion, the reaction mixture was filtered through a sintered glass filter to remove the white crystalline byproduct, dicyclohexylurea. The filtrate was sequentially washed with a diluted hydrochloric acid solution (H_2_O: conc. HCl = 4:1, *v/v*), followed by sodium carbonate solution (3%, *w/v*), and brine. The organic layer was dried over anhydrous Na_2_SO_4_, and the solvent was evaporated under reduced pressure. The residue was dissolved in ethyl acetate, and the flask was placed in an ice bath for approximately 30 min to ensure complete crystallization of any remaining dicyclohexylurea. The mixture was filtered again to remove the residual dicyclohexylurea. The filtrate was concentrated under vacuum to remove the ethyl acetate. The resulting amide product was dissolved in dichloromethane and washed sequentially with the sodium carbonate solution and water. The organic layer was again dried over anhydrous Na_2_SO_4_ and concentrated under reduced pressure to afford the purified compound. All compounds were fully characterized by ^1^H and ^13^C NMR, as well as HRMS, with the corresponding spectra provided in the Supplementary Information file (Figures S1–S15).

**3a** 2-(4-isobutylphenyl)-1-(4-(2,3,4-trimethoxybenzyl)piperazin-1-yl)propan-1-one

Yellow oil, yield of 98% (0.445 g) R_f_ = 0.31 (Et_2_O/CH_3_OH = 2:0.1 *v/v*) ^1^H NMR (400 MHz, DMSO) 1H NMR (400 MHz, DMSO) δ 7.18–7.03 (m, 4H), 6.89 (d, J = 8.5 Hz, 1H), 6.73 (d, J = 8.6 Hz, 1H), 4.02 (q, J = 6.7 Hz, 1H), 3.76 (s, 3H), 3.72 (s, 6H), 3.41–3.30 (m, 4H), 3.27 (d, J = 5.8 Hz, 2H), 2.40 (d, J = 7.2 Hz, 2H), 2.33 (td, J = 8.4, 6.7, 2.9 Hz, 1H), 2.26–2.18 (m, 1H), 2.17–2.10 (m, 1H), 1.81 (m, 1H), 1.77 (m, 1H), 1.26 (d, J = 6.8 Hz, 3H), 0.85 (d, J = 6.6 Hz, 6H). 13C NMR (101 MHz, DMSO) δ 171.80, 153.00, 152.44, 142.29, 140.06, 139.66, 129.61, 127.38, 125.11, 123.77, 107.97, 61.32, 60.70, 56.21, 56.14, 52.90, 52.67, 44.66, 41.39, 30.09, 22.62, 21.01. HRMS electrospray ionization (ESI) m/z calcd for [M + H]+ C_27_H_39_N_2_O_4_^+^ = 455.2905, found 455.2913 (mass error ∆m = 1.76 ppm).

**3b** 2-(3-benzoylphenyl)-1-(4-(2,3,4-trimethoxybenzyl)piperazin-1-yl)propan-1-one

Pale yellow oil, yield of 86% (0.4350 g), R_f_ = 0.50 (Et_2_O/CH_3_OH = 2:0.1, *v/v*). ^1^H NMR (400 MHz, DMSO) δ 7.75–7.65 (m, 3H), 7.64–7.46 (m, 6H), 6.92 (d, J = 8.5 Hz, 1H), 6.73 (d, J = 8.6 Hz, 1H), 4.24 (q, J = 6.7 Hz, 1H), 3.75 (s, 3H), 3.72 (s, 3H), 3.71 (s, 3H), 3.35 (s, 4H), 3.32 (s, 2H), 2.42–2.24 (m, 2H), 2.16 (m, 1H), 1.88 (m, 1H), 1.30 (d, J = 6.8 Hz, 3H). ^13^C NMR (101 MHz, DMSO) δ 196.15, 171.39, 153.02, 152.46, 143.21, 142.27, 137.77, 137.48, 133.23, 132.19, 130.06, 129.41, 129.03, 128.77, 128.43, 125.22, 123.64, 108.00, 61.35, 60.71, 56.20, 56.08, 53.03, 52.61, 41.29, 20.87. HRMS electrospray ionization (ESI) m/z calcd for [M + H]^+^ C_30_H_35_N_2_O_5_^+^ = 503.2541, found 503.2550 (mass error ∆m = 1.79 ppm).

**3c** 2-(6-methoxynaphthalen-2-yl)-1-(4-(2,3,4-trimethoxybenzyl)piperazin-1-yl)propan-1-one

Yellow oil, yield of 97% (0.4676 g), R_f_ = 0.55 (Et_2_O/CH_3_OH = 2:0.1, v/v). ^1^H NMR (400 MHz, DMSO) δ 7.76 (dd, J = 8.8, 2.4 Hz, 2H), 7.65 (s, 1H), 7.36 (dd, J = 8.5, 1.8 Hz, 1H), 7.28 (d, J = 2.6 Hz, 1H), 7.14 (dd, J = 8.9, 2.5 Hz, 1H), 6.88 (d, J = 8.5 Hz, 1H), 6.70 (d, J = 8.5 Hz, 1H), 4.19 (q, J = 6.7 Hz, 1H), 3.86 (s, 3H), 3.75 (s, 3H), 3.70 (s, 3H), 3.66 (s, 3H), 3.35 (s, 4H), 3.26 (s, 2H), 2.37–2.28 (m, 1H), 2.18 (ddt, J = 14.4, 10.1, 5.6 Hz, 2H), 1.86–1.77 (m, 1H), 1.35 (d, J = 6.7 Hz, 3H). ^13^C NMR (101 MHz, DMSO) δ 171.78, 157.54, 152.97, 152.41, 142.24, 137.85, 133.51, 129.50, 128.99, 127.58, 126.62, 125.80, 125.14, 123.70, 119.13, 107.96, 106.21, 61.25, 60.69, 56.20, 56.05, 55.62, 52.98, 52.69, 41.61, 20.99. HRMS electrospray ionization (ESI) m/z calcd for [M + H]^+^ C_28_H_35_N_2_O_5_^+^ = 479.2541, found 479.2551 (mass error ∆m = 2.09 ppm).

**3d** 2-(2-fluoro-[1,1’-biphenyl]-4-yl)-1-(4-(2,3,4-trimethoxybenzyl)piperazin-1-yl)propan-1-one

Colorless oil, yield of 93% (0.4442 g), R_f_ = 0.62 (Et_2_O/CH_3_OH = 2:0.1, *v/v*). ^1^H NMR (400 MHz, DMSO) δ 7.62–7.51 (m, 2H), 7.50–7.43 (m, 3H), 7.43–7.33 (m, 1H), 7.24–7.15 (m, 2H), 6.93 (d, J = 8.5 Hz, 1H), 6.73 (d, J = 8.6 Hz, 1H), 4.20 (q, J = 6.8 Hz, 1H), 3.76 (s, 3H), 3.73 (s, 3H), 3.72 (s, 3H), 3.52–3.45 (m, 2H), 3.34 (d, J = 1.9 Hz, 4H), 2.29 (dtd, J = 17.2, 11.6, 5.2 Hz, 3H), 2.04 (dt, J = 10.4, 4.7 Hz, 1H), 1.32 (d, J = 6.8 Hz, 3H). ^13^C NMR (101 MHz, DMSO) δ 171.29, 159.42 (d, ^1^*J*_C-F_ = 246.2 Hz), 153.02, 152.48, 144.60, 142.28, 135.35, 131.30 (d, ^3^*J*_C-F_ = 3.7 Hz), 129.18, 129.07, 128.23, 126.88, 125.23, 124.22, 123.69, 115.39 (d, ^2^*J*_C-F_ = 23.1 Hz), 107.97, 61.35, 60.70, 56.09, 53.09, 52.66, 45.62, 42.11, 20.68. HRMS electrospray ionization (ESI) *m/z* calcd for [M + H]^+^ C_29_H_34_FN_2_O_4_^+^ = 493.2498, found 493.2507 (mass error ∆m = 1.82 ppm).

**3e** 2-(6-chloro-9H-carbazol-2-yl)-1-(4-(2,3,4-trimethoxybenzyl)piperazin-1-yl)propan-1-one

White crystals (m.p. 96–98 °C), yield of 92% (0.2502 g), R_f_ = 0.48 (Et_2_O/CH_3_OH = 2:0.1, *v/v*). ^1^H NMR (400 MHz, DMSO) δ 11.34 (s, 1H), 8.16 (dt, J = 2.2, 0.6 Hz, 1H), 8.08 (dt, J = 8.1, 0.7 Hz, 1H), 7.48 (dd, J = 8.6, 0.6 Hz, 1H), 7.40–7.33 (m, 2H), 7.07 (dd, J = 8.1, 1.5 Hz, 1H), 6.86 (d, J = 8.6 Hz, 1H), 6.68 (d, J = 8.6 Hz, 1H), 4.22 (q, J = 6.7 Hz, 1H), 3.74 (s, 3H), 3.68 (s, 3H), 3.64 (s, 3H), 3.52–3.42 (m, 2H), 3.35 (s, 2H), 3.28–3.19 (m, 2H), 2.32 (m, 1H), 2.19 (m, 2H), 1.81 (m, 1H), 1.35 (d, J = 6.7 Hz, 3H). ^13^C NMR (101 MHz, DMSO) δ 171.90, 152.97, 152.41, 142.23, 141.36, 141.16, 138.78, 125.53, 125.14, 124.12, 123.72, 123.34, 121.34, 120.67, 120.07, 119.07, 112.77, 109.87, 107.95, 61.24, 60.67, 56.18, 55.37, 52.97, 52.64, 42.23, 21.55. HRMS electrospray ionization (ESI) m/z calcd for [M + H]^+^ C_29_H_33_ClN_3_O_4_^+^ = 522.2155, found 522.2167 (mass error ∆m = 2.30 ppm).

### 3.3. Biological Activity

#### 3.3.1. Hydrogen Peroxide Scavenging Activity (HPSA)

The Manolov et al. approach was used to evaluate a capacity to scavenge hydrogen peroxide [46]. A 43 mM solution of H_2_O_2_ was prepared in a potassium phosphate buffer solution (0.2 M, pH 7.4). The analysis of the samples was carried out as follows: in test tubes, 0.6 mL H_2_O_2_ (43 mM), 1 mL sample/standard with different concentrations (20–1000 µg/mL), and 2.4 mL potassium phosphate buffer solution were mixed. The mixture was stirred and incubated in the dark for 10 min at 37 °C. Absorbance was measured at 230 nm with a spectrophotometer (CamSpec M508, Leeds, UK) against a blank solution containing the phosphate buffer and H_2_O_2_ without the sample. Ascorbic acid and quercetin were used as standards. The percentage HPSA of the samples was evaluated by comparing with a blank sample, and was calculated using the following formula:

(1) $$I,\%(HPSA)=\left[\frac{A_{blank}-(A_{TS}-A_{CS})}{A_{blank}}\right]\times 100$$

where A_blank_ is the absorbance of the blank sample, A_CS_ is the absorbance of the control sample, and A_TS_ is the absorbance of the test sample.

#### 3.3.2. Hydroxyl Radical Scavenging Activity (HRSA)

The hydroxyl radical-scavenging activity of the samples was measured according to the method of Luo et al. [47]. In this system, hydroxyl radicals were generated by the Fenton reaction. Hydroxyl radicals could oxidize Fe^2+^ into Fe^3+^, and only Fe^2+^ could be combined with 1,10-phenanthroline to form a red compound (1,10-phenanthroline-Fe^2+^) with a maximum absorbance at 536 nm. The concentration of the hydroxyl radical was reflected by the degree of decolorization of the reaction solution. In test tubes 1 mL of 1,10-phenanthroline solution (0.75 mM), 2.0 mL of phosphate buffer saline (0.2 M, pH 7.40), and 1 mL of the samples/standard were added and mixed homogeneously. A total of 1.0 mL of the FeSO_4_ solution (0.75 mM) was then pipetted into the mixture. The reaction was initiated by adding 1.0 mL H_2_O_2_ (0.03% v/v). After incubation at 37°C for 60 min in a water bath, the absorbance of the reaction mixture was measured at 536 nm against reagent blank. The reaction mixture without any antioxidant was used as the negative control, and without H_2_O_2_ was used as the blank. The hydroxyl radical scavenging activity (HRSA) was calculated by the following formula:

(2) $$I,\%(HRSA)=\left[\frac{A_{S}-A_{n}}{A_{b}-A_{n}}\right]\times 100$$

where A_s_, A_n_, and A_b_ were the absorbance values determined at 536 nm of the sample, the negative control, and the blank after reaction, respectively. Quercetin was used as the positive control.

#### 3.3.3. Inhibition of Albumin Denaturation (IAD)

The in vitro analysis of the anti-inflammatory activity was evaluated by measuring the inhibition of albumin denaturation (IAD). The analysis was carried out according to the method of Manolov et al. [48] with minor modifications [49]. The experiment was conducted using human albumin. A 1% albumin solution was prepared in distilled water at pH 7.4. Test samples and standards were initially dissolved in 1 mL of DMSO, then supplemented with 1% Tween 80 in PBS to achieve a final stock solution concentration of 1000 μg/mL. Subsequently, a series of working solutions at varying concentrations (20–500 μg/mL) in 1% Tween 80/PBS were prepared. The reaction mixture consisted of 2 mL of the test sample or standard at varying concentrations and 1 mL of 1% albumin. The mixture was incubated at 37 °C for 15 min, followed by heating at 70 °C for 15 min in a water bath. After cooling, the turbidity was measured at 660 nm using a spectrophotometer (CamSpec M508, Leeds, UK). Ibuprofen served as the standard. The experiment was conducted in triplicate. The percentage inhibition of albumin denaturation (IAD) was calculated against the control. The control sample consisted of albumin at the same concentration dissolved in distilled water.

(3) $$\%IAD=\left[\frac{A_{blank}-A_{sample}}{A_{blank}}\right]\times 100$$

#### 3.3.4. Molecular Docking

To investigate the interaction of ligands **3a**–**e** with human serum albumin (HSA), molecular docking studies were performed using both AutoDock Vina 1.1.2 (ADV) and AutoDock 4.2 (AD) [50,51], because they rely on different computational algorithms, offering complementary insights that help to minimize the likelihood of false-positive results in docking predictions [52]. The three-dimensional structure of HSA was retrieved from the Protein Data Bank (PDB ID: 7JWN, https://www.rcsb.org/, accessed on 27 June 2025), as was used in our previous papers [53,54]. For each ligand were generated both R and S stereoisomers using Avogadro 1.2.0 [55].

The preparation of human serum albumin (HSA) targeted in the present study followed the standard protocols previously established by our group, including removal of the co-crystallized ligand, addition of polar hydrogen atoms, and assignment of charges [53,56]. Preparations for both the ligands and the macromolecule were completed using AutoDock Tools 1.5.6 [51].

Molecular docking was performed targeting four key binding sites in HSA: Sudlow site I (subdomain IIA)—center coordinates x = 30.62, y = 25.50, z = 12.43; Sudlow site II (subdomain IIIA)—center coordinates x = 5.95, y = 18.22, z = 21.06; site III—center coordinates x = 30.15, y = 26.98, z = 37.99; and the cleft region—center coordinates x = 20.89, y = 21.74, z = 22.43. These sites were selected based on their established relevance in drug binding, while other reported sites were considered less significant for ligand interaction with albumin. The search space for each binding pocket was defined as a cubic grid, with 20 Å sides for AutoDock Vina (ADV) and 54 Å for AutoDock (AD) with 0.375 Å spacing [53,57,58,59,60,61,62].

To enhance reproducibility, ADV was configured to generate 20 binding poses per ligand per site, while AD generated 200 poses per site to facilitate a clustering analysis, using a root mean square deviation (RMSD) threshold of 2 Å. The docking outcomes were analyzed and visualized using UCSF Chimera 1.10.2 [63].

#### 3.3.5. Molecular Dynamics Simulations

For each compound, the highest-affinity conformation identified by AutoDock Vina molecular docking across all investigated binding sites was used to construct a chimeric complex with human serum albumin (HSA). For each enantiomer, at least one conformation demonstrating strong and stable binding during molecular dynamics (MD) simulations was selected. In cases where a particular enantiomer’s conformation at a given binding site did not exhibit sufficient stability (maximum 0.5 nm threshold the average of the RMSD of the ligand heavy atoms during the simulation), an alternative conformation of the same enantiomer was chosen from a different binding site, based on descending docking affinities, and used further for molecular dynamics simulation.

All simulations were conducted using GROMACS 2023, employing the CHARMM36 force field and the TIP4P water model within an orthorhombic simulation box [64,65,66]. Ligands were parameterized using the CGenFF protocol, and the systems were assembled, charge-neutralized, and subjected to energy minimization in line with previously established methodologies [67,68,69].

Simulations were run for 100 nanoseconds to monitor the time-dependent structural behavior of each complex. The computational setup was equipped with an AMD Ryzen 9 7900 and an NVIDIA RTX 3060 GPU, with CUDA 12.8 for accelerated performance under Debian Linux 12. Visualizations were carried out using VMD 1.9.4 [70]. The trajectory analysis was performed using the built-in functions of GROMACS.

#### 3.3.6. Determination of Lipophilicity as RM Values

The lipophilicity of the trimetazidine–profen derivatives was determined following the procedure described by Pontiki and Hadjipavlou-Litina [37].

#### 3.3.7. Statistical Analysis

All experiments were performed in triplicate, and the data are presented as mean ± standard deviation (SD). A significance threshold of *p* < 0.05 was applied for all statistical evaluations. Statistical analyses were conducted using SPSS software (version 19.0; SPSS Inc., Chicago, IL, USA). Differences between mean activity values were assessed using one-way ANOVA, followed by Duncan’s post hoc test to determine statistically significant groupings.

## 4. Conclusions

Five new trimetazidine–profen hybrids were synthesized and confirmed by NMR and MS. Among them, compound **3c** showed the best antioxidant (HRSA IC_50_ = 71.13 µg/mL) and anti-inflammatory (IC_50_ = 108.58 µg/mL) activities. Lipophilicity tests (R_M_) indicated moderate values, suggesting good membrane permeability, with compounds **3c** and **3d** having the most balanced profiles. Docking studies revealed that S-enantiomers generally bond stronger to HSA, with compound **3c** interacting across Sudlow sites II and III, while compound **3d[S]** anchored most tightly at site II. Molecular dynamics (100 ns) confirmed stable binding, with compound **3c** maintaining consistent interactions and compound **3d[S]** showing high stability at site II. These findings highlight compound **3c** as the most promising lead for further development as a dual-action therapeutic.

## Figures and Tables

**Table 1:** The binding affinity of compounds **3a**–**e** to the four targeted HSA sites—AutoDock Vina (ΔG kcal/mol).

Compound	Enantiomer	Cleft	Sudlow I	Sudlow II	Site III
**3a**	R	−9.2	−8.2	−10.1	−10.4
	S	−9.7	−8.2	−10.2	−10.1
**3b**	R	−10.0	−9.4	−11.0	−12.0
	S	−10.5	−9.1	−11.4	−11.8
**3c**	R	−9.8	−8.5	−10.4	−11.2
	S	−10.3	−8.5	−11.2	−11.5
**3d**	R	−9.9	−8.7	−11.1	−10.4
	S	−10.5	−8.7	−11.5	−11.0
**3e**	R	−10.2	−8.9	−10.5	−11.2
	S	−10.7	−8.0	−10.8	−10.3

**Table 2:** The binding affinity of compounds **3a**–**e** to the four targeted HSA sites—AutoDock ΔG kcal/mol).

Compound	Enantiomer	Cleft		Sudlow I		Sudlow II		Site III	
		ΔG	%C	ΔG	%C	ΔG	%C	ΔG	%C
**3a**	R	−8.56	26	−8.59	2	−10.45	38	−11.70	48
	S	−9.17	36	−7.56	4	−10.04	24	−10.98	24
**3b**	R	−9.26	16	−9.33	6	−11.41	28	−12.53	22
	S	−10.22	28	−8.48	6	−11.39	10	−12.03	24
**3c**	R	−9.42	14	−7.63	6	−11.46	44	−11.38	44
	S	−9.68	32	−8.44	8	−11.44	36	−11.28	54
**3d**	R	−9.05	20	−7.90	4	−11.67	48	−11.41	32
	S	−9.73	32	−8.52	4	−11.80	26	−11.33	36
**3e**	R	−10.00	26	−8.41	2	−11.96	20	−12.08	44
	S	−9.72	24	−8.86	8	−11.86	10	−12.21	16

%C: percent of conformations in the same 2Å RMSD cluster of atom coordinates.

**Table 3:** The average root mean square deviation of the heavy atoms of the ligand (nm).

System Evaluated	Cleft	Sudlow I	Sudlow II	Site III
**apo + 3a[R]**	-	-	-	0.49
**apo + 3a[S]**	-	-	0.29	-
**apo + 3b[R]**	-	-	-	0.44
**apo + 3b[S]**	-	-	-	0.32
**apo + 3c[R]**	-	-	0.28 ^b^	0.76 ^a^
**apo + 3c[S]**	-	-	0.32 ^b^	0.92 ^a^
**apo + 3d[R]**	-	-	0.26	-
**apo + 3d[S]**	-	-	0.23	-
**apo + 3e[R]**	-	-	0.26 ^b^	0.92 ^a^
**apo + 3e[S]**	-	-	0.33	-

-, not tested; a, unstable; b, tested due to the instability of another ligand–protein complex on another site.

**Table 4:** The root mean square deviation of the backbone of HSA (nm).

System Evaluated	Cleft	Sudlow I	Sudlow II	Site III
**apo + 3a[R]**	-	-	-	0.35
**apo + 3a[S]**	-	-	0.42	-
**apo + 3b[R]**	-	-	-	0.19
**apo + 3b[S]**	-	-	-	0.19
**apo + 3c[R]**	-	-	0.17	unstable complex
**apo + 3c[S]**	-	-	0.22	unstable complex
**apo + 3d[R]**	-	-	0.29	-
**apo + 3d[S]**	-	-	0.22	-
**apo + 3e[R]**	-	-	0.19	unstable complex
**apo + 3e[S]**	-	-	0.19	-
**apo**	0.38			

-, not tested.

**Table 5:** The radius of gyration of HSA (nm).

System Evaluated	Cleft	Sudlow I	Sudlow II	Site III
**apo + 3a[R]**	-	-	-	2.74
**apo + 3a[S]**	-	-	2.73	-
**apo + 3b[R]**	-	-	-	2.81
**apo + 3b[S]**	-	-	-	2.77
**apo + 3c[R]**	-	-	2.72	unstable complex
**apo + 3c[S]**	-	-	2.77	unstable complex
**apo + 3d[R]**	-	-	2.79	-
**apo + 3d[S]**	-	-	2.75	-
**apo + 3e[R]**	-	-	2.76	unstable complex
**apo + 3e[S]**	-	-	2.76	-
**apo**	2.78			

-, not tested.

**Table 6:** The average number of hydrogen bonds between the ligand and HSA (no/ns).

System Evaluated	Cleft	Sudlow I	Sudlow II	Site III
**apo + 3a[R]**	-	-	-	0.91
**apo + 3a[S]**	-	-	0.66	-
**apo + 3b[R]**	-	-	-	2.27
**apo + 3b[S]**	-	-	-	0.08
**apo + 3c[R]**	-	-	0.29	unstable complex
**apo + 3c[S]**	-	-	0.37	unstable complex
**apo + 3d[R]**	-	-	0.00	-
**apo + 3d[S]**	-	-	0.61	-
**apo + 3e[R]**	-	-	0.00	unstable complex
**apo + 3e[S]**	-	-	0.98	-

-, not tested.

**Table 7:** Prediction of acute rat toxicity using GUSAR software.

Compound	Rat LD_50_ [mg/kg]			
	**IP**	**IV**	**Oral**	**SC**
**1**	259.8^4a^	35.05^3a^	732.4^4a^	553.6^4a^
**3a**	485.1^4a^	20.35^3a^	878.2^4a^	232.3^4b^
**3b**	634.4^5a^	41.53^4a^	1299^4a^	738.1^4a^
**3c**	477.6^4a^	58.81^4a^	1003^4a^	833.2^4a^
**3d**	535.6^5a^	50.74^4a^	722^4a^	346.8^4a^
**3e**	532.7^5b^	53.57^4a^	892.8^4a^	731^4a^

IP–intraperitoneal, IV–intravenous, SC–subcutaneous. Applicability domain: a-compound falls in the applicability domain of models; b-compound does not fall in the applicability domain of models. The superscript numbers show the acute rodent toxicity classification by the OECD (Organization for Economic Co-operation and Development). The OECD classifies the chemicals into five categories, where 1 is highly toxic and 5 is practically non-toxic.

## Data Availability

Data are contained within the article and the supplementary materials.

## Supplementary Materials

The following supporting information can be downloaded at: https://www.mdpi.com/article/10.3390/ph18091251/s1, Figure S1: ^1^H-NMR spectrum of compound **3a**; Figure S2: ^1^H-NMR spectrum of compound **3b**; Figure S3: ^1^H-NMR spectrum of compound **3c**; Figure S4: ^1^H-NMR spectrum of compound **3d**; Figure S5: ^1^H-NMR spectrum of compound **3e**; Figure S6: ^13^C-NMR spectrum of compound **3a**; Figure S7: ^13^C-NMR spectrum of compound **3b**; Figure S8: ^13^C-NMR spectrum of compound **3c**; Figure S9: ^13^C-NMR spectrum of compound **3d**; Figure S10: ^13^C-NMR spectrum of compound **3e**; Figure S11: ESI-HRMS of compound **3a**; Figure S12: ESI-HRMS of compound **3b**; Figure S13: ESI-HRMS of compound **3c**; Figure S14: ESI-HRMS of compound **3d**; Figure S15: ESI-HRMS of compound **3e**; Figure S16: Analysis of the evolution of the complex of **3a[R]** docked in site III of HSA; Figure S17: Analysis of the evolution of the complex of **3a[S]** docked in Sudlow site II of HAS; Figure S18: Analysis of the evolution of the complex of **3b[R]** docked in site III of HAS; Figure S19: Analysis of the evolution of the complex of **3b[S]** docked in site III of HSA; Figure S20: Analysis of the evolution of the complex of **3c[R]** docked in site III of HSA; Figure S21: Analysis of the evolution of the complex of **3c[S]** docked in site III of HSA; Figure S22: Analysis of the evolution of the complex of **3d[R]** docked in Sudlow site II of HSA; Figure S23: Analysis of the evolution of the complex of **3d[S]** docked in Sudlow site II of HAS; Figure S24: Analysis of the evolution of the complex of **3e[R]** docked in site III of HSA; Figure S25: Analysis of the evolution of the complex of **3e[S]** docked in Sudlow site II of HSA; Table S1: Antioxidant activity (HPSA, HRSA) and anti-inflammatory activity, evaluated by inhibition albumin denaturation (IAD) of trimetazidine derivatives (**3a**–**e**).
