# Supplementary material for: Trimetazidine–Profen Hybrid Molecules: Synthesis, Chemical Characterization, and Biological Evaluation of Their Racemates
Source: Pharmaceuticals (Basel). 2025 Aug 23;18(9):1251. doi: 10.3390/ph18091251 (PMC12472577; doi:10.3390/ph18091251)
Supplement: Supplementary file 1 [file pharmaceuticals-18-01251-s001.zip › pharmaceuticals-3820474-supplementary.pdf]

Supplementary Materials:

Trimetazidine–Profen Hybrid Molecules: Synthesis, Chemical Characterization, and Biological Evaluation of Their Racemates

Diyana Dimitrova, Stanimir Manolov, Iliyan Ivanov, Dimitar Bojilov, Nikol Dimova, Gabriel Marc, Smaranda Oniga and Ovidiu Oniga

Table of Contents:

Figure S1. ^{1}H-NMR spectrum of compound **3a** page **3**

Figure S2. ^{1}H-NMR spectrum of compound **3b** page **4**

Figure S3. ^{1}H-NMR spectrum of compound **3c** page **5**

Figure S4. ^{1}H-NMR spectrum of compound **3d** page **6**

Figure S5. ^{1}H-NMR spectrum of compound **3e** page **7**

Figure S6. ^{13}C-NMR spectrum of compound **3a** page **8**

Figure S7. ^{13}C-NMR spectrum of compound **3b** page **9**

Figure S8. ^{13}C-NMR spectrum of compound **3c** page **10**

Figure S9. ^{13}C-NMR spectrum of compound **3d** page **11**

Figure S10. ^{13}C-NMR spectrum of compound **3e** page **12**

Figure S11. ESI-HRMS of compound **3a** page **13**

Figure S12. ESI-HRMS of compound **3b** page **14**

Figure S13. ESI-HRMS of compound **3c** page **15**

Figure S14. ESI-HRMS of compound **3d** page **16**

Figure S15. ESI-HRMS of compound **3e** page **17**

Figure S16. Analysis of the evolution of the complex of **3a[R]** docked in site III of HSA. Page **18**

Figure S17. Analysis of the evolution of the complex of **3a[S]** docked in Sudlow site II of HSA. Page **19**

Figure S18. Analysis of the evolution of the complex of **3b[R]** docked in site III of HSA. Page **20**

Figure S19. Analysis of the evolution of the complex of **3b[S]** docked in site III of HSA. Page **21**

Figure S20. Analysis of the evolution of the complex of **3c[R]** docked in site III of HSA. Page **22**

Figure S21. Analysis of the evolution of the complex of **3c[S]** docked in site III of HSA. Page **23**

Figure S22. Analysis of the evolution of the complex of **3d[R]** docked in Sudlow site II of HSA. Page **24**

Figure S23. Analysis of the evolution of the complex of **3d[S]** docked in Sudlow site II of HAS. Page **25**

Figure S24. Analysis of the evolution of the complex of **3e[R]** docked in site III of HSA. Page **26**

Figure S25. Analysis of the evolution of the complex of **3e[S]** docked in Sudlow site II of HSA. Page **27**

Table S1. Antioxidant activity (HPSA, HRSA) and anti-inflammatory activity, evaluated by inhibition albumin denaturation (IAD) of trimetazidine derivatives (**3a-e**). Page **28**

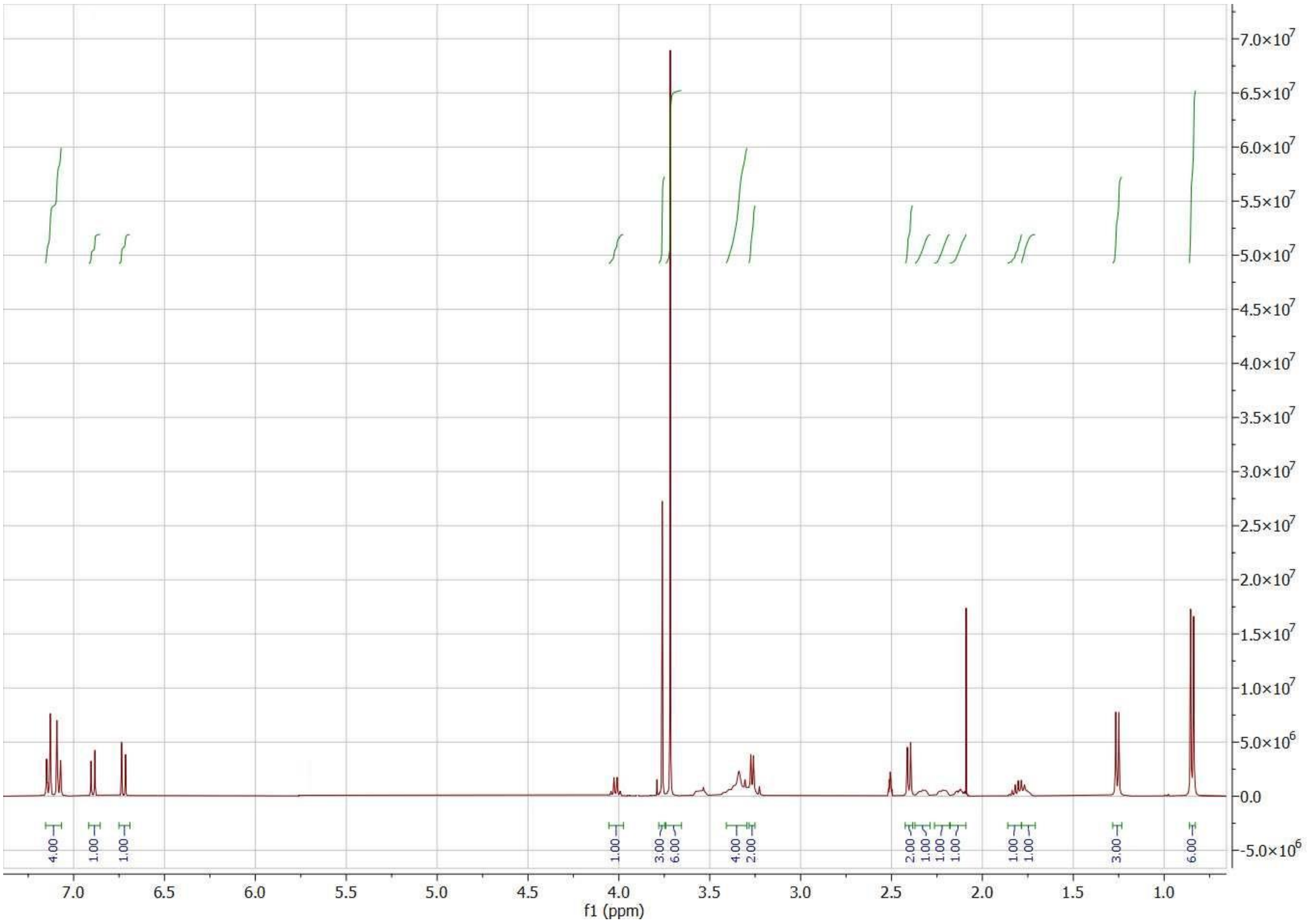

Figure S1. ^{1}H-NMR spectrum of compound **3a**.

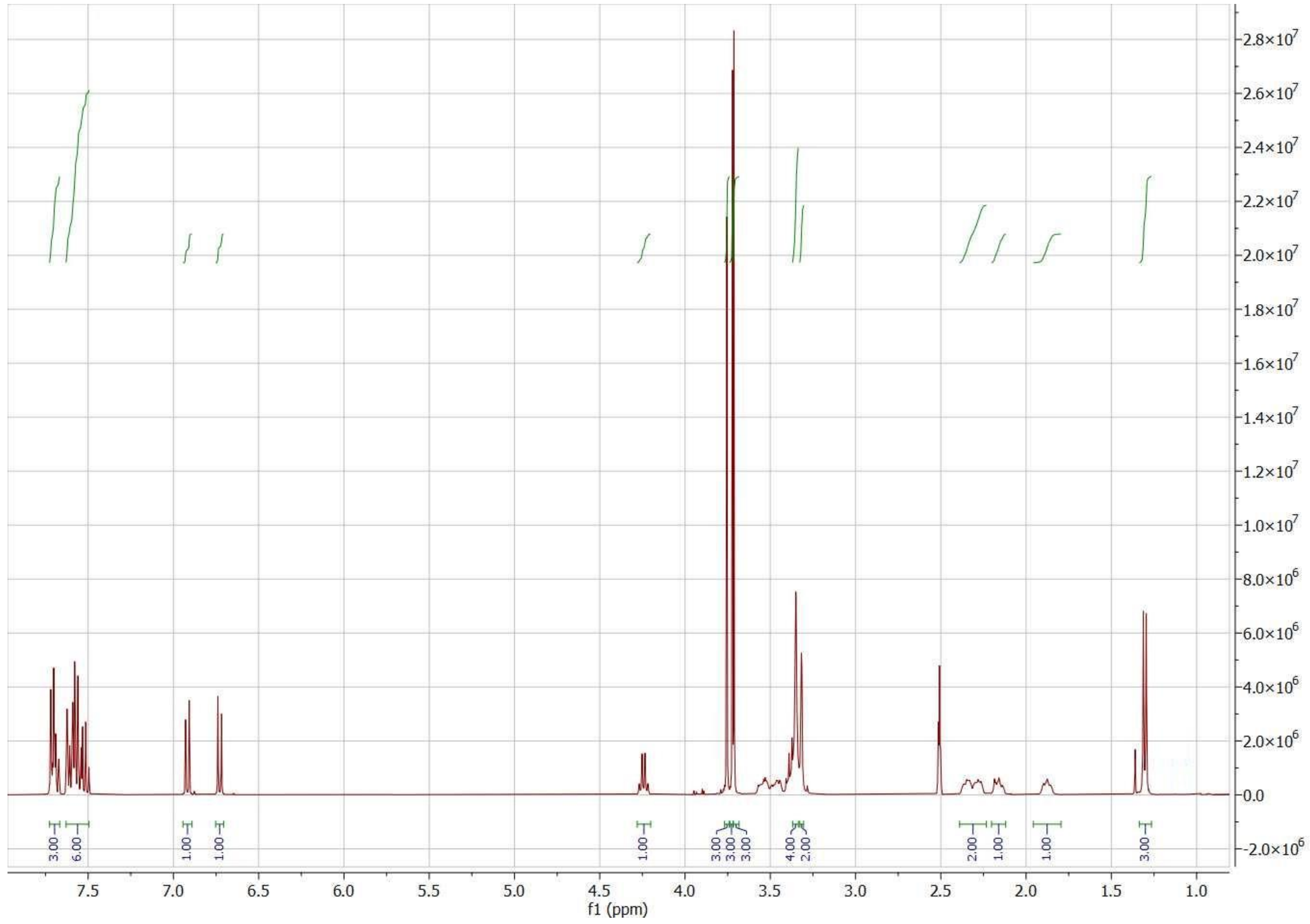

Figure S2. ^{1}H-NMR spectrum of compound **3b**.

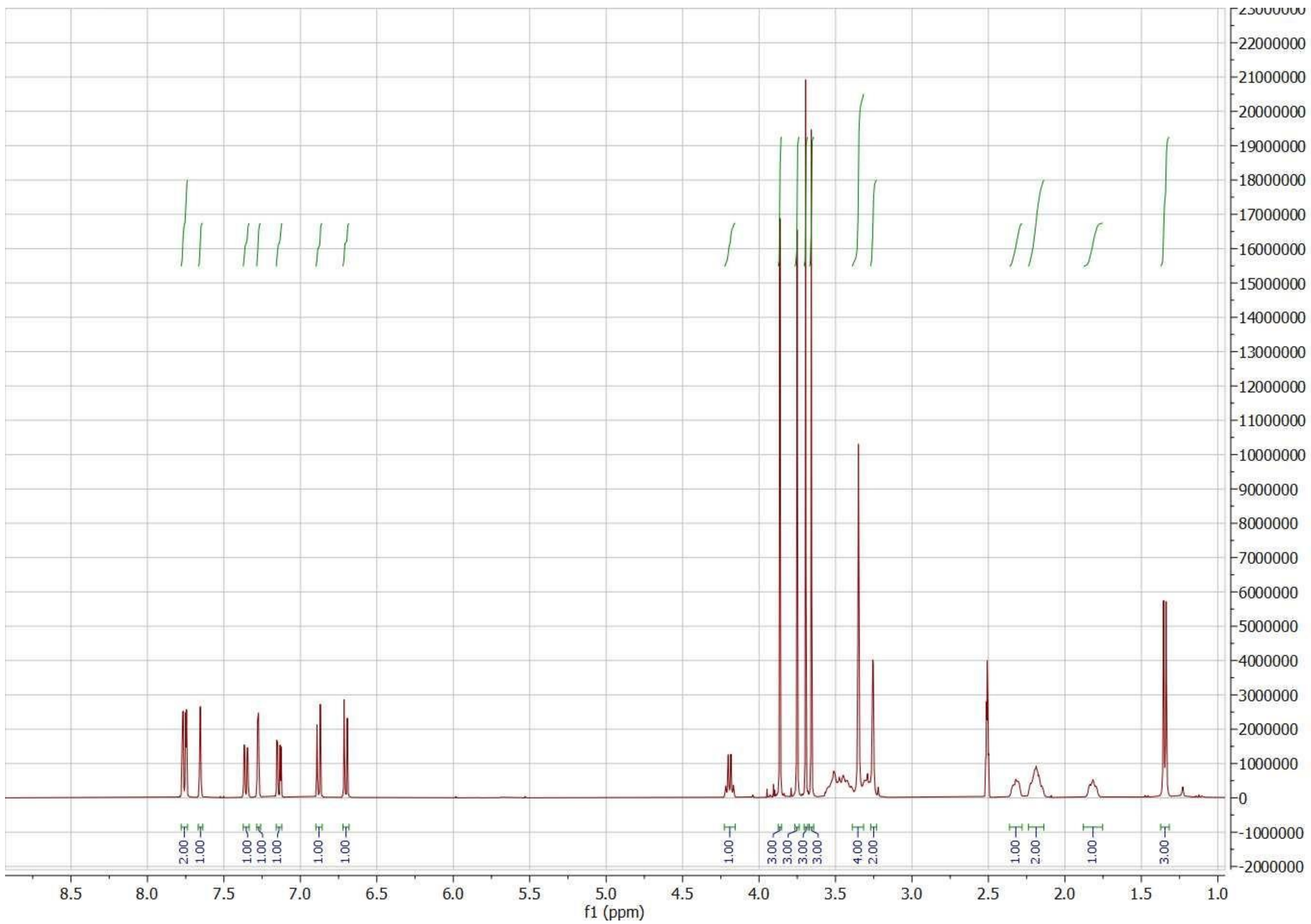

Figure S3. ¹H-NMR spectrum of compound **3c**.

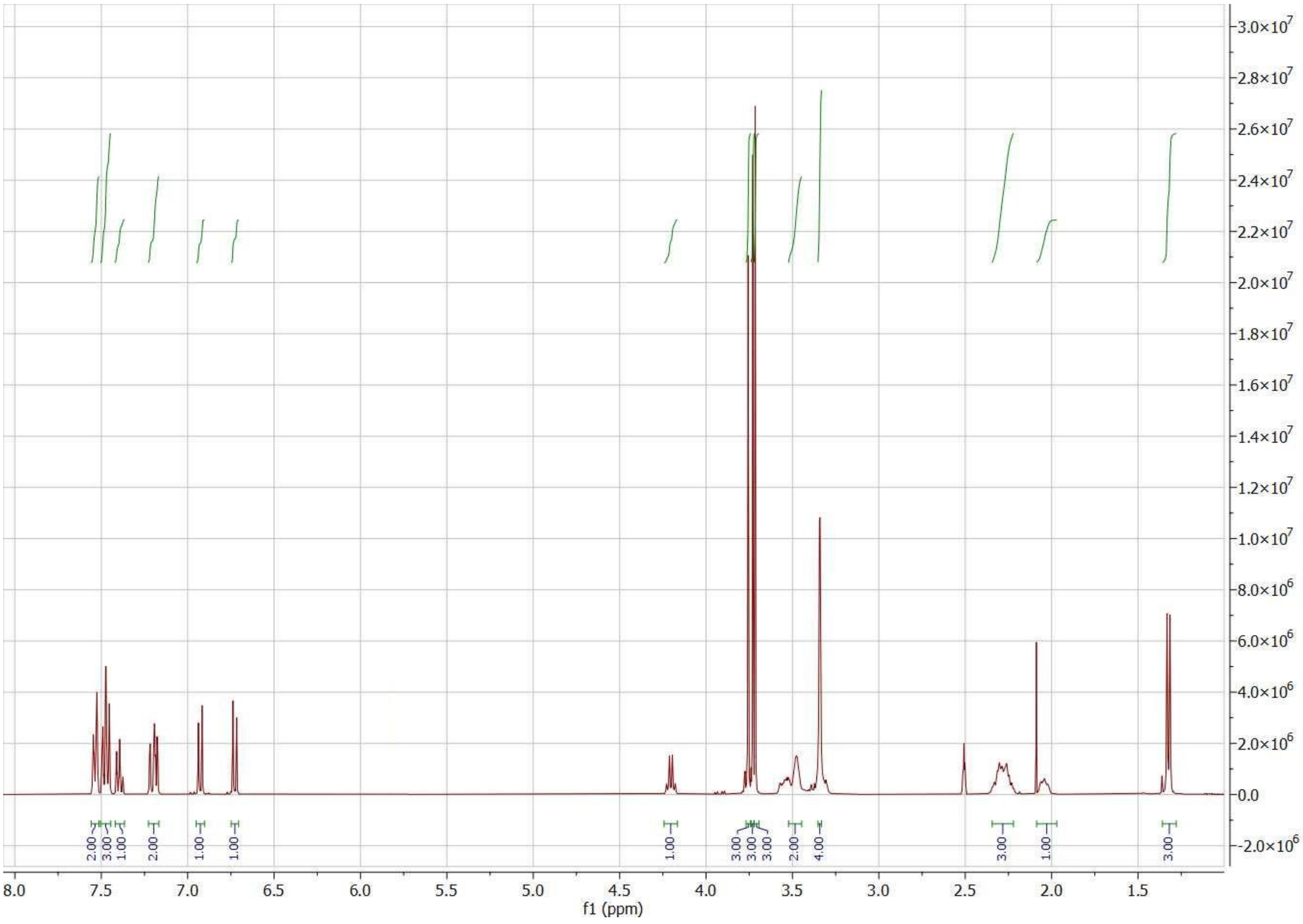

Figure S4. ^{1}H-NMR spectrum of compound **3d**.

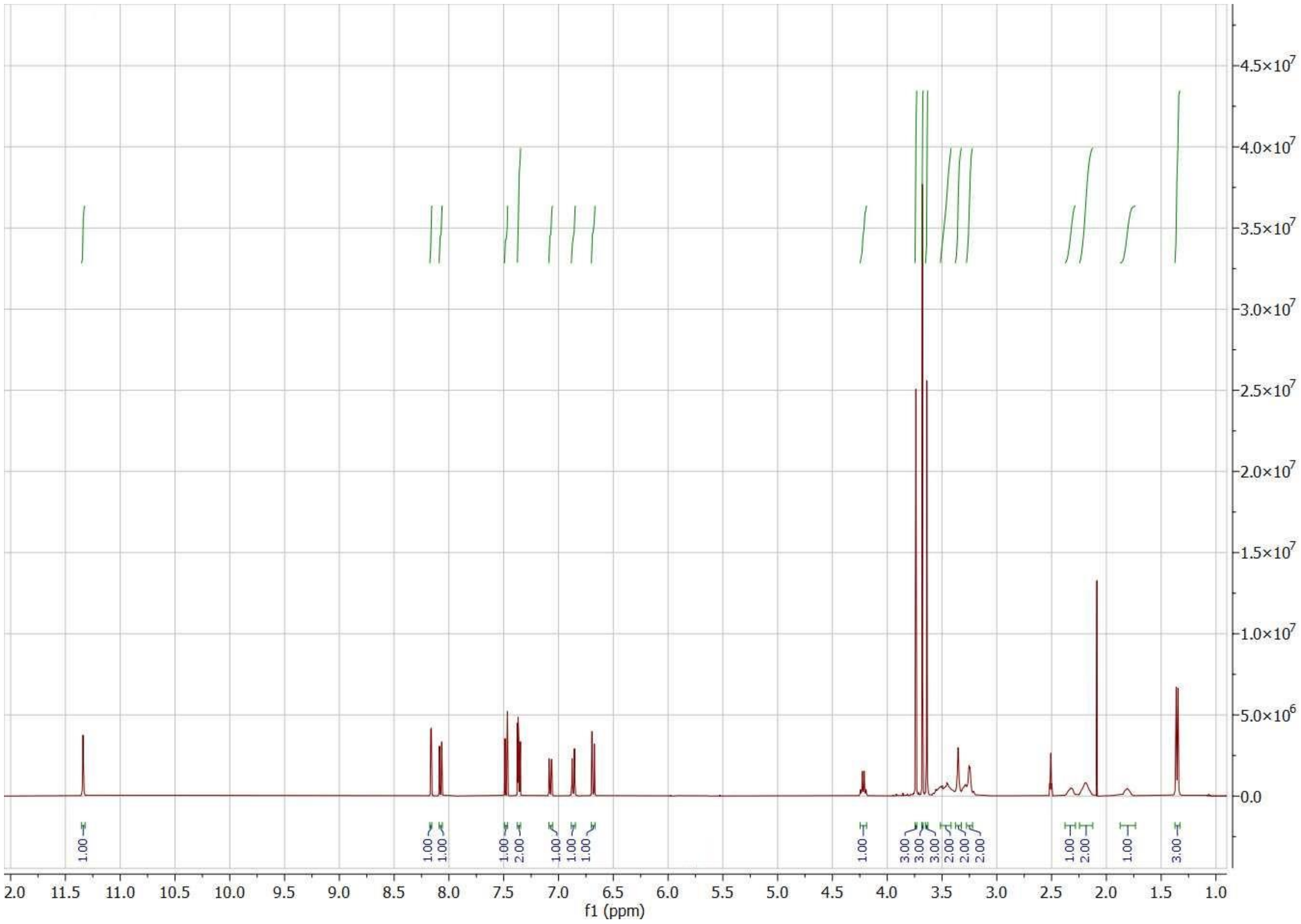

Figure S5. ^{1}H-NMR spectrum of compound **3e**.

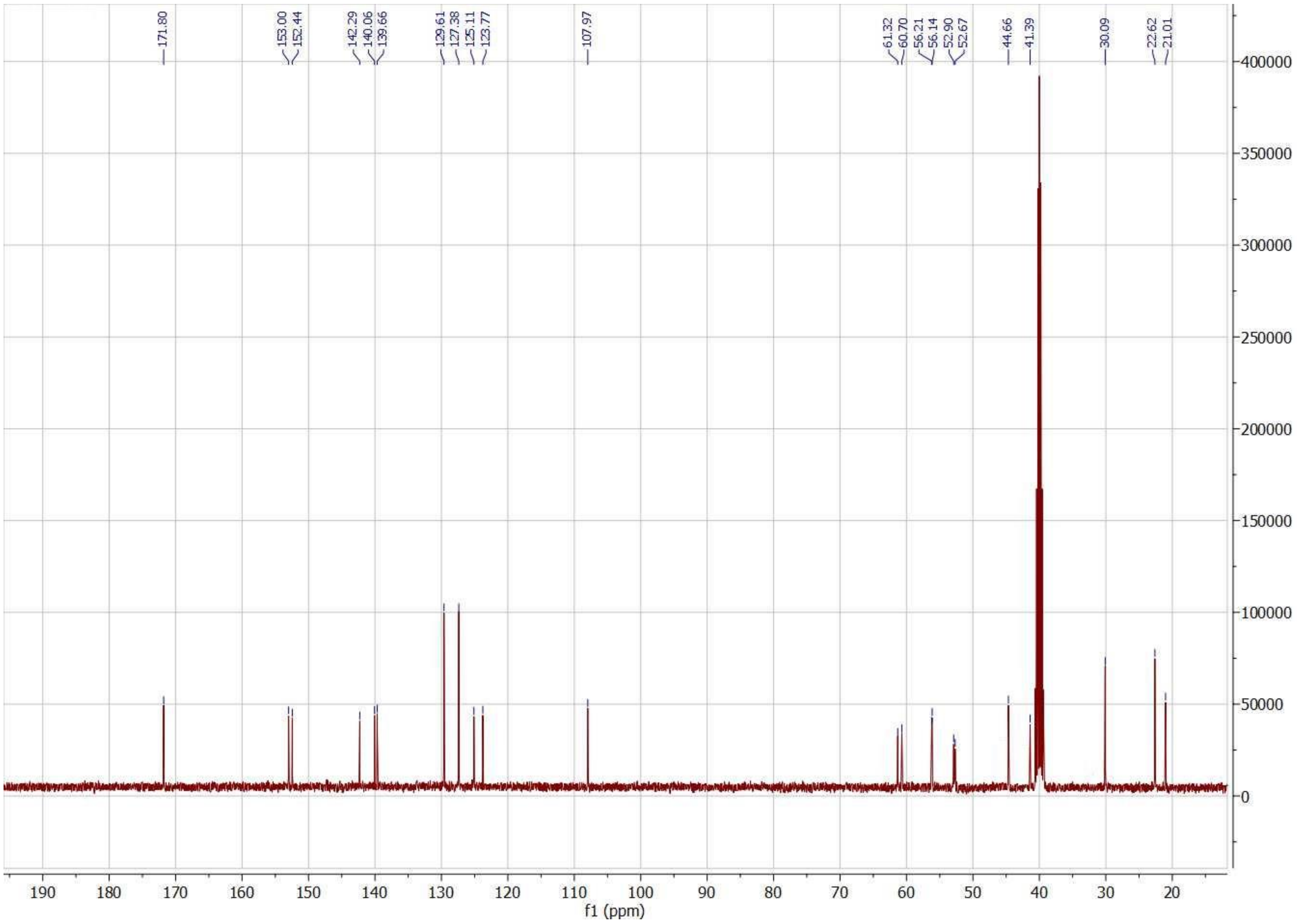

Figure S6. ^{13}C-NMR spectrum of compound **3a**.

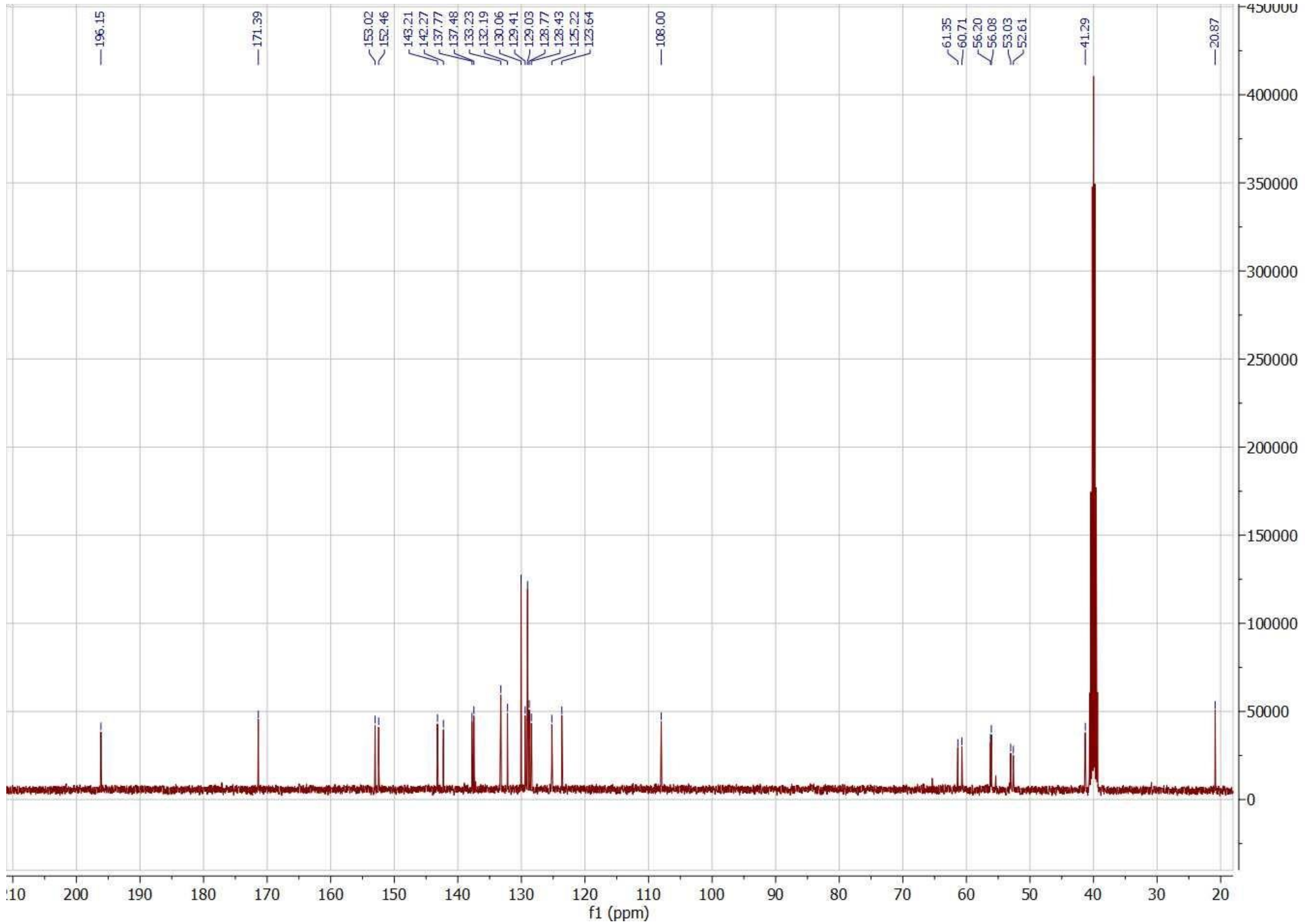

Figure S7. ^{13}C-NMR spectrum of compound **3b**.

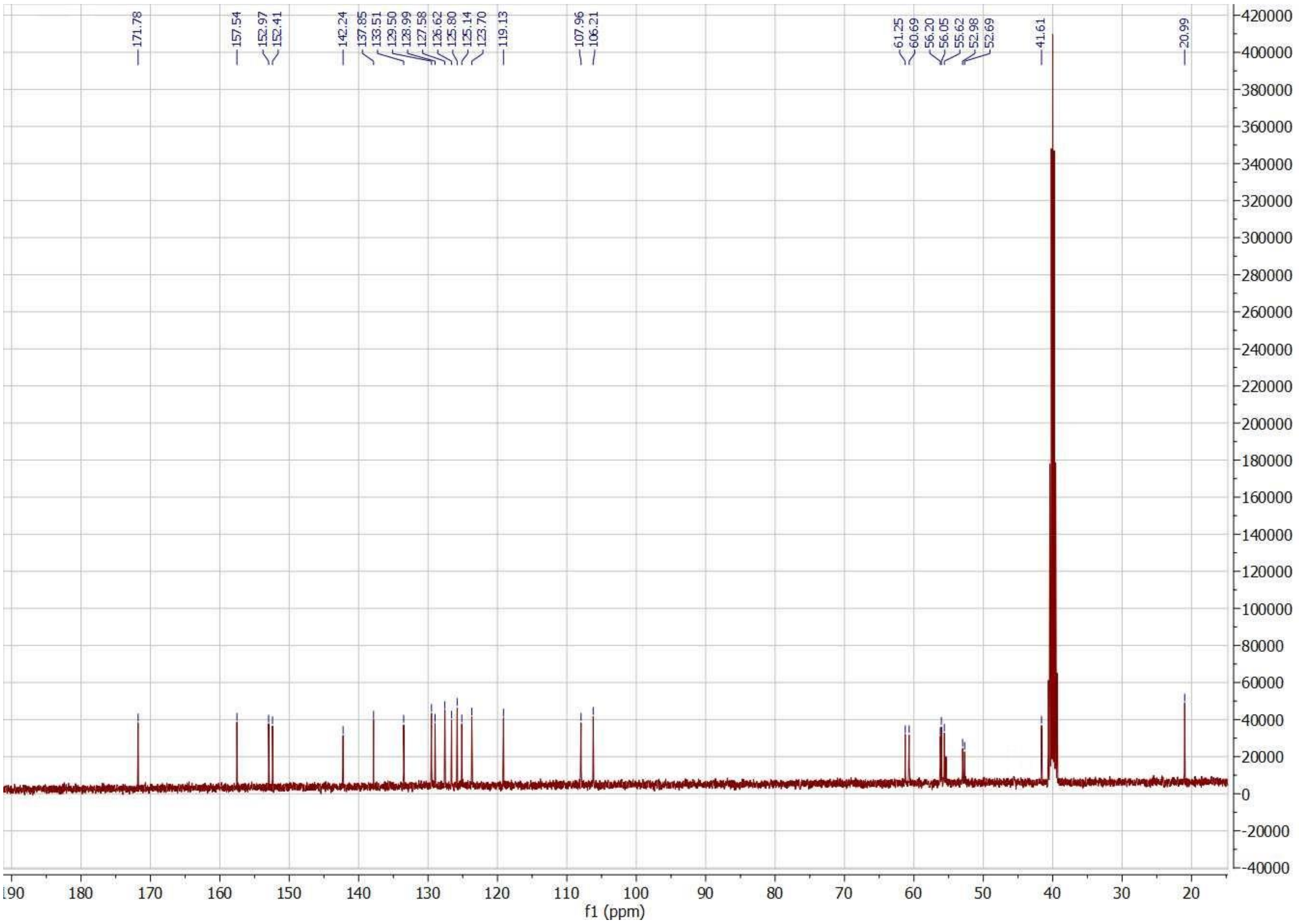

Figure S8. ^{13}C-NMR spectrum of compound **3c**.

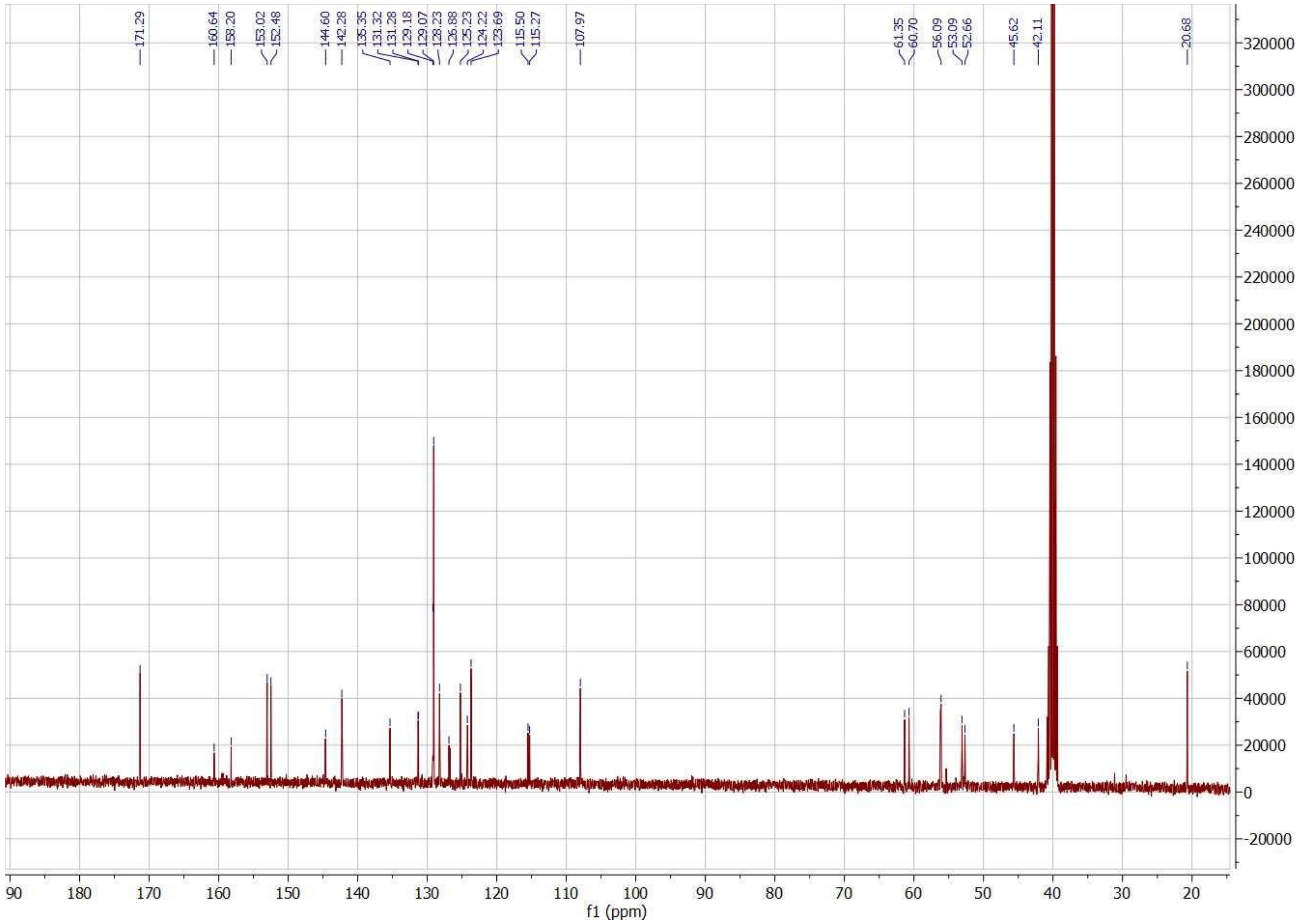

Figure S9. ^{13}C-NMR spectrum of compound **3d**.

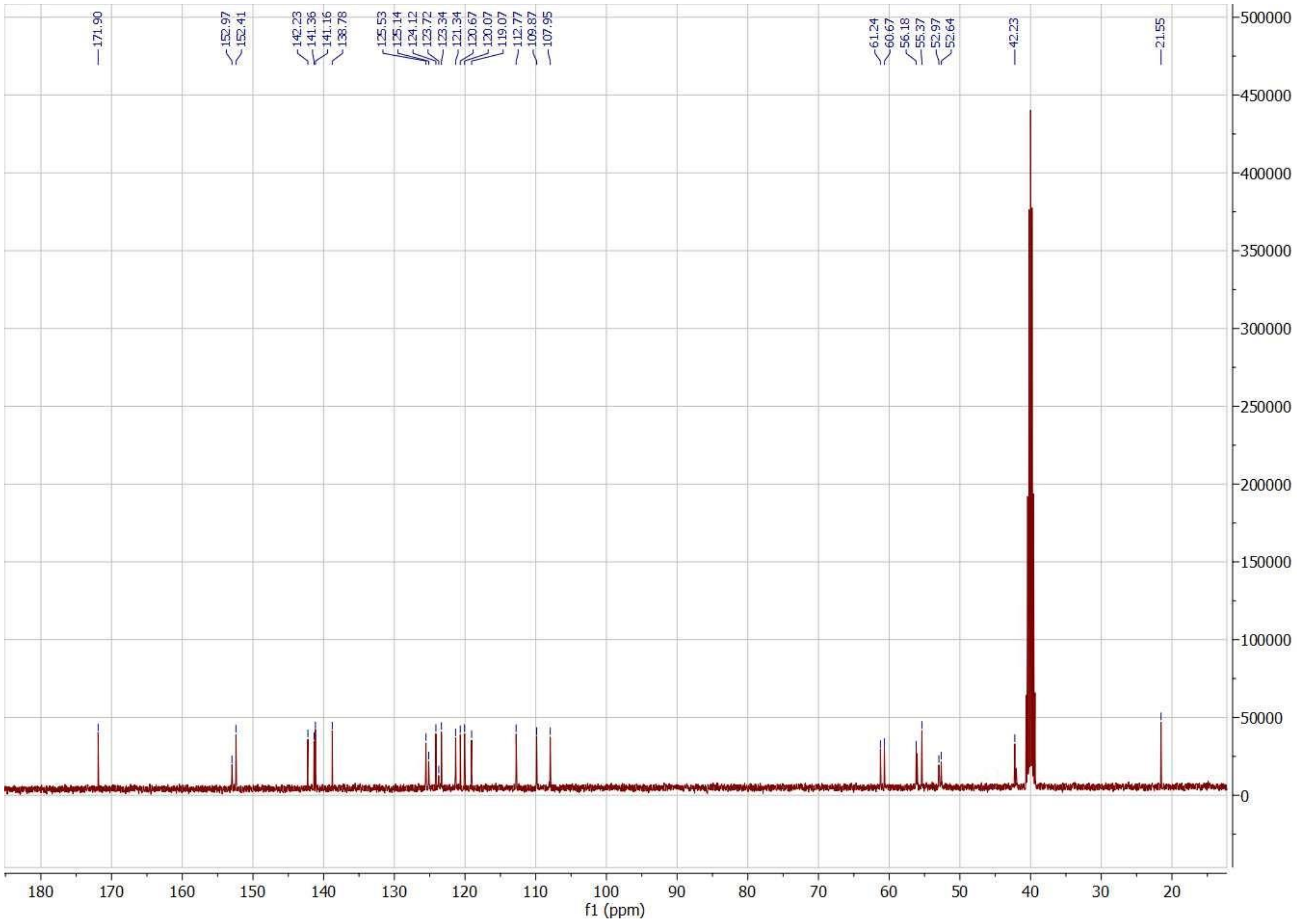

Figure S10. ^{13}C-NMR spectrum of compound **3e**.

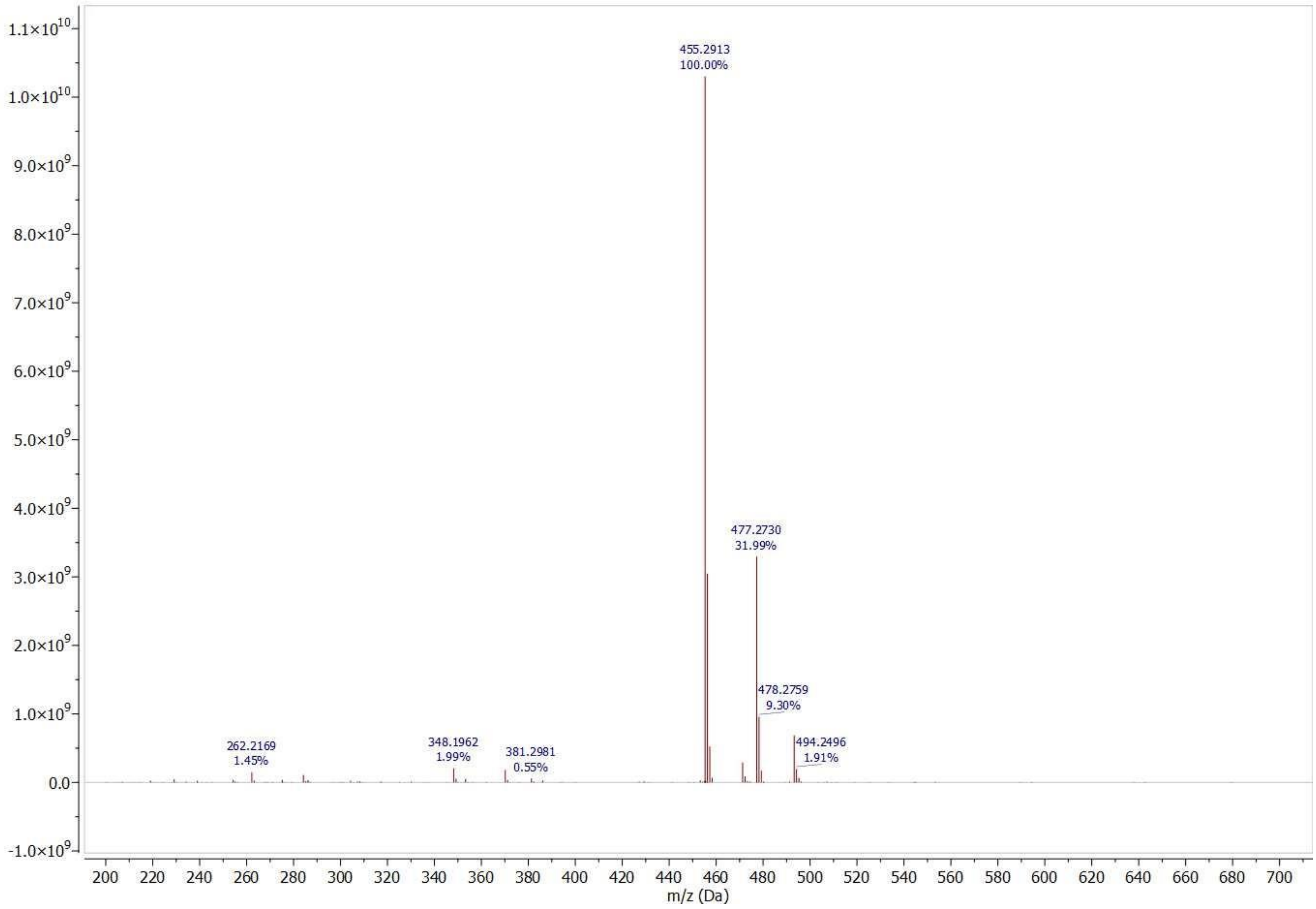

Figure S11. ESI-HRMS of compound **3a**.

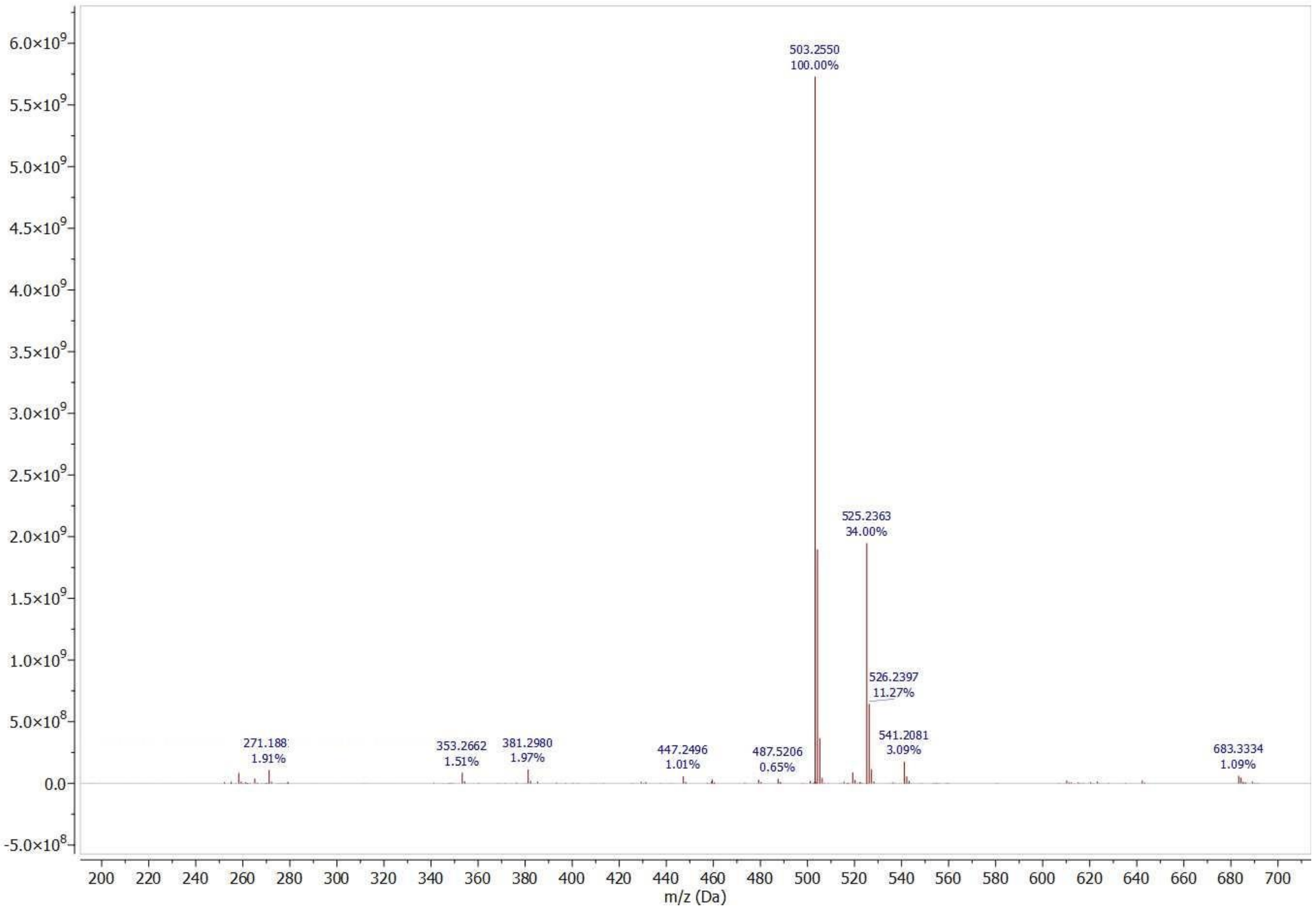

Figure S12. ESI-HRMS of compound **3b**.

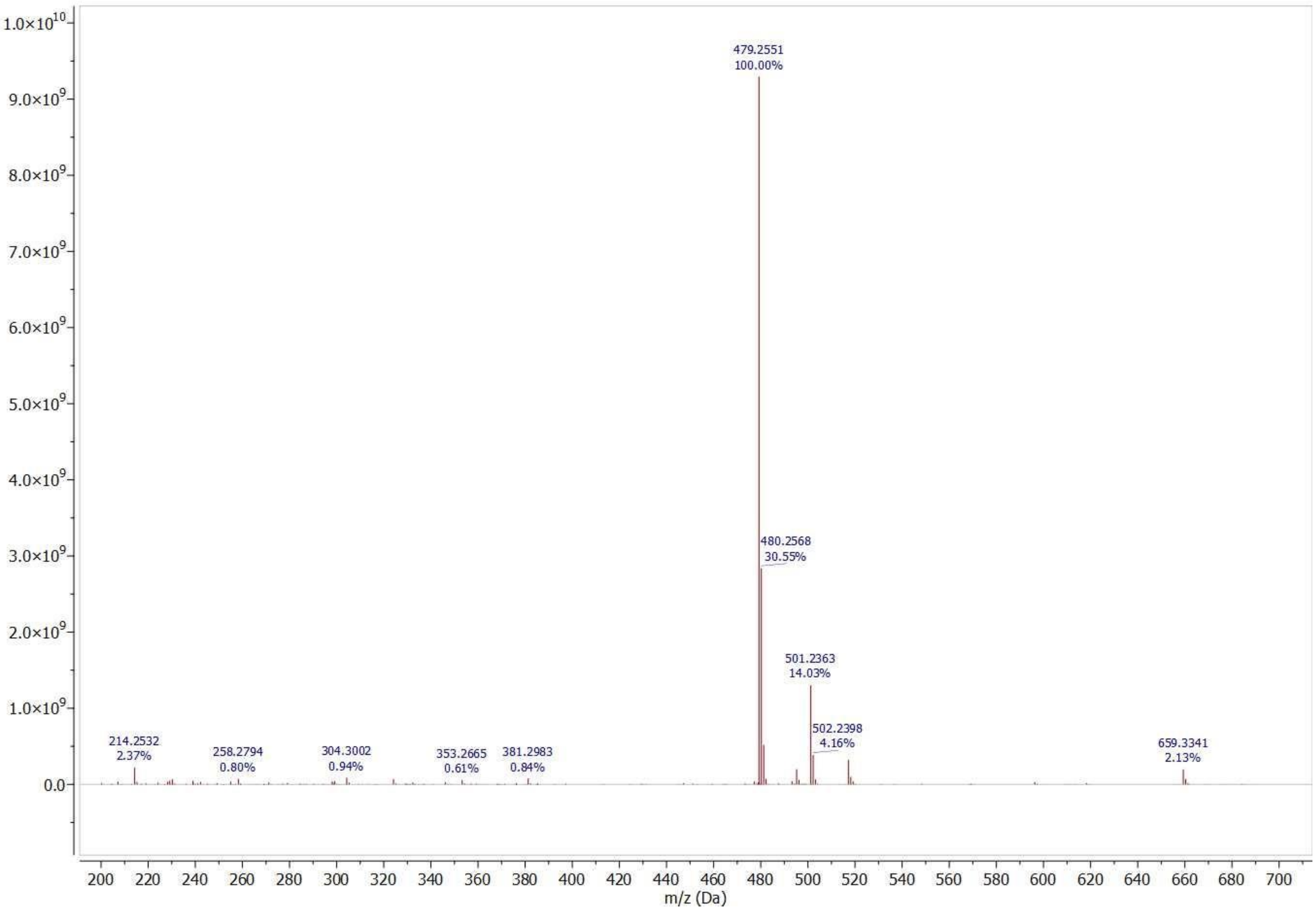

Figure S13. ESI-HRMS of compound **3c**.

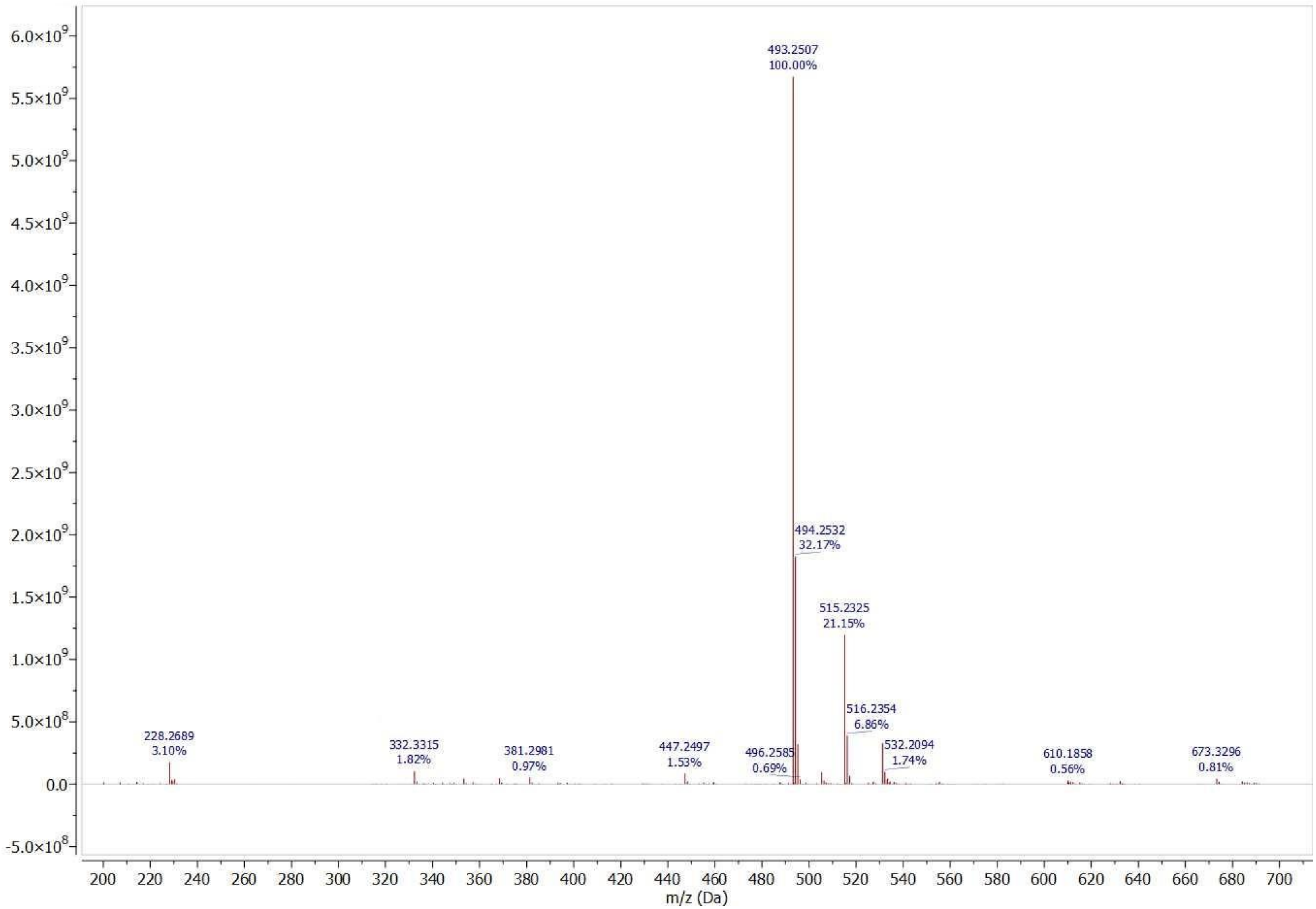

Figure S14. ESI-HRMS of compound **3d**.

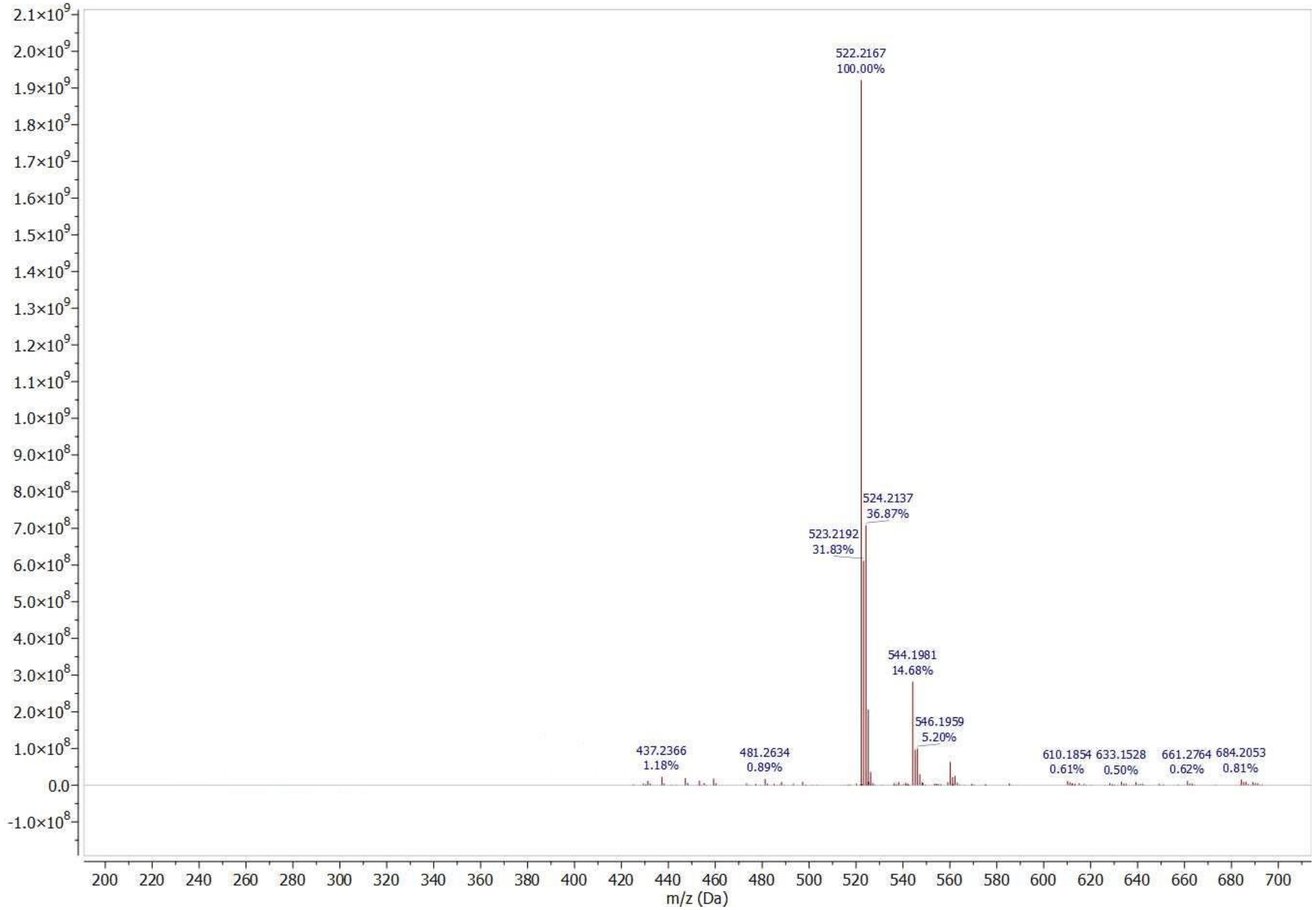

Figure S15. ESI-HRMS of compound **3e**.

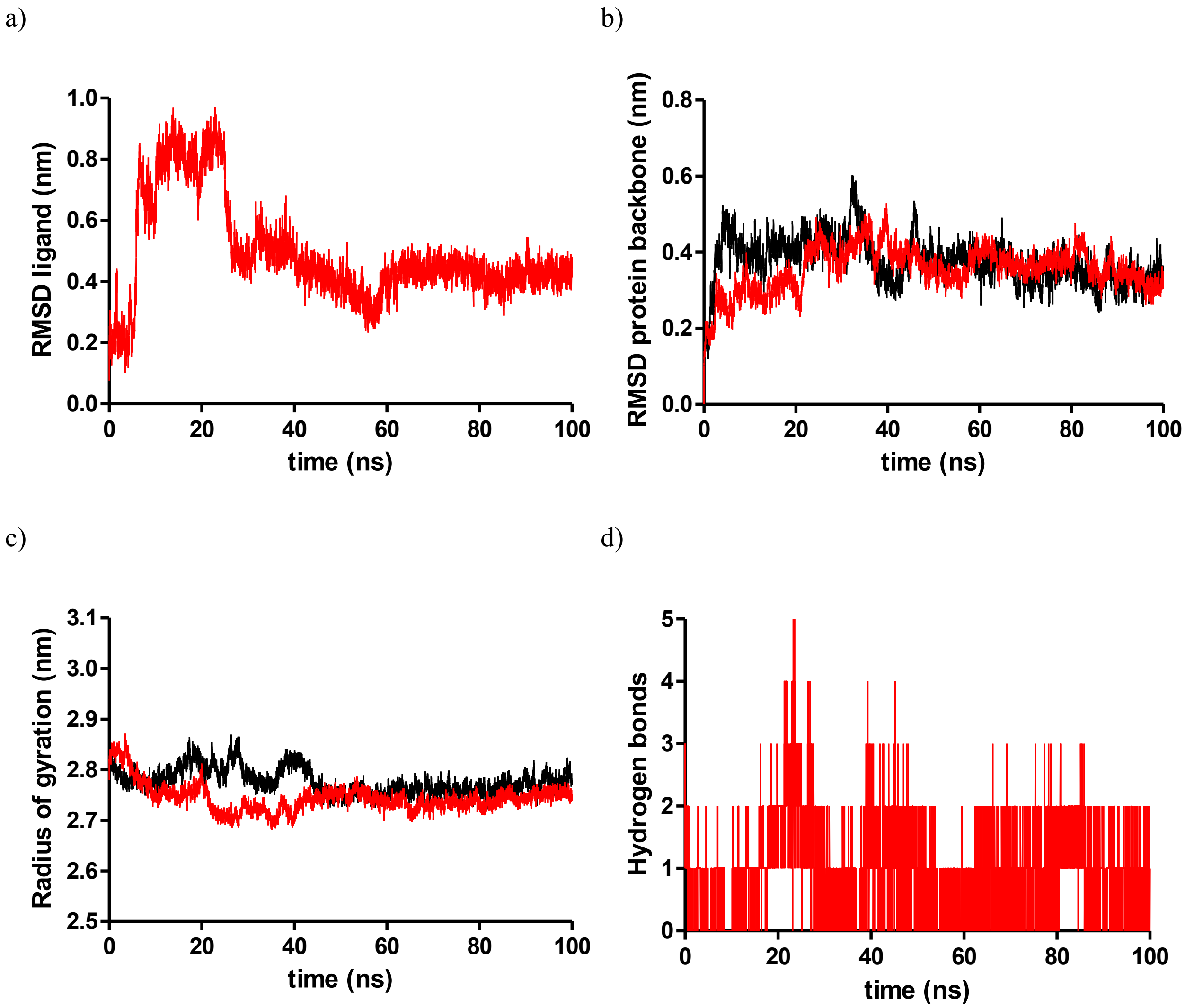

Figure S16. Analysis of the evolution of the complex of **3a[R]** docked in site III of HSA: (a) RMSD of heavy atoms of the ligand; (b) RMSD of HSA backbone apo (black) and in complex with the ligand (red); (c) RG of HSA backbone apo (black) and in complex with the ligand (red); (d) hydrogen bonds between the ligand and HAS.

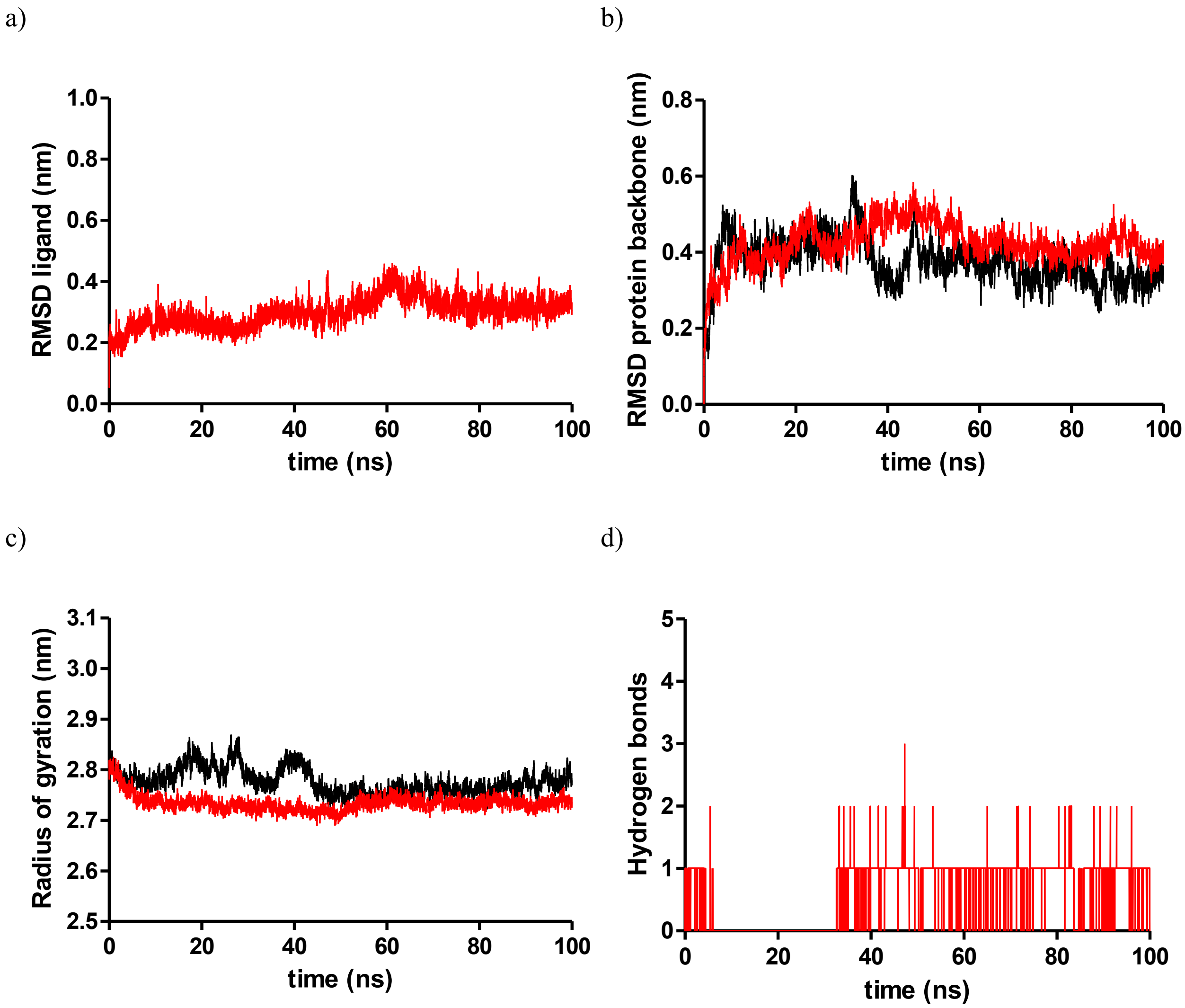

Figure S17. Analysis of the evolution of the complex of **3a[S]** docked in Sudlow site II of HSA: (a) RMSD of heavy atoms of the ligand; (b) RMSD of HSA backbone apo (black) and in complex with the ligand (red); (c) RG of HSA backbone apo (black) and in complex with the ligand (red); (d) hydrogen bonds between the ligand and HAS.

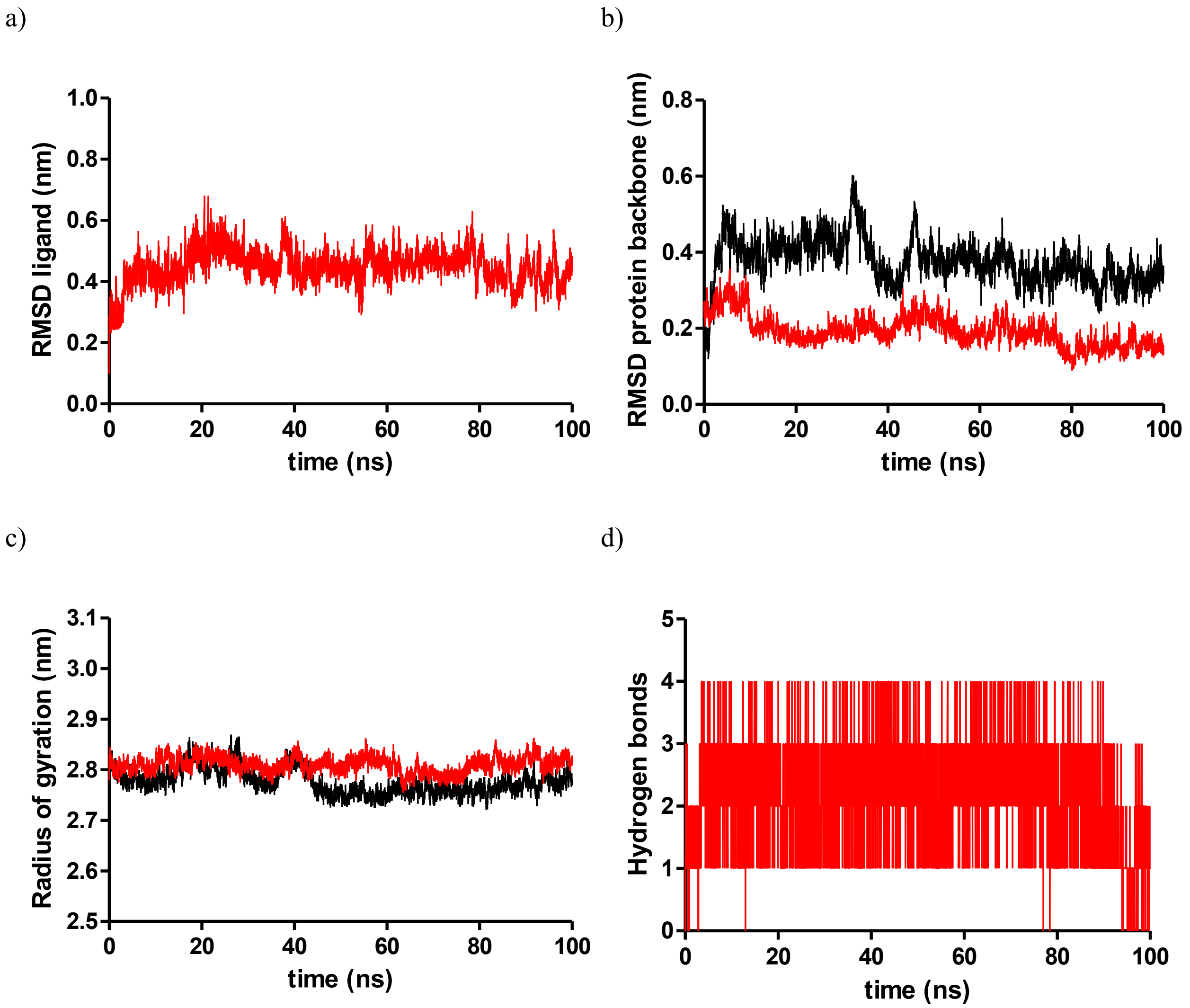

Figure S18. Analysis of the evolution of the complex of **3b[R]** docked in site III of HSA: (a) RMSD of heavy atoms of the ligand; (b) RMSD of HSA backbone apo (black) and in complex with the ligand (red); (c) RG of HSA backbone apo (black) and in complex with the ligand (red); (d) hydrogen bonds between the ligand and HAS.

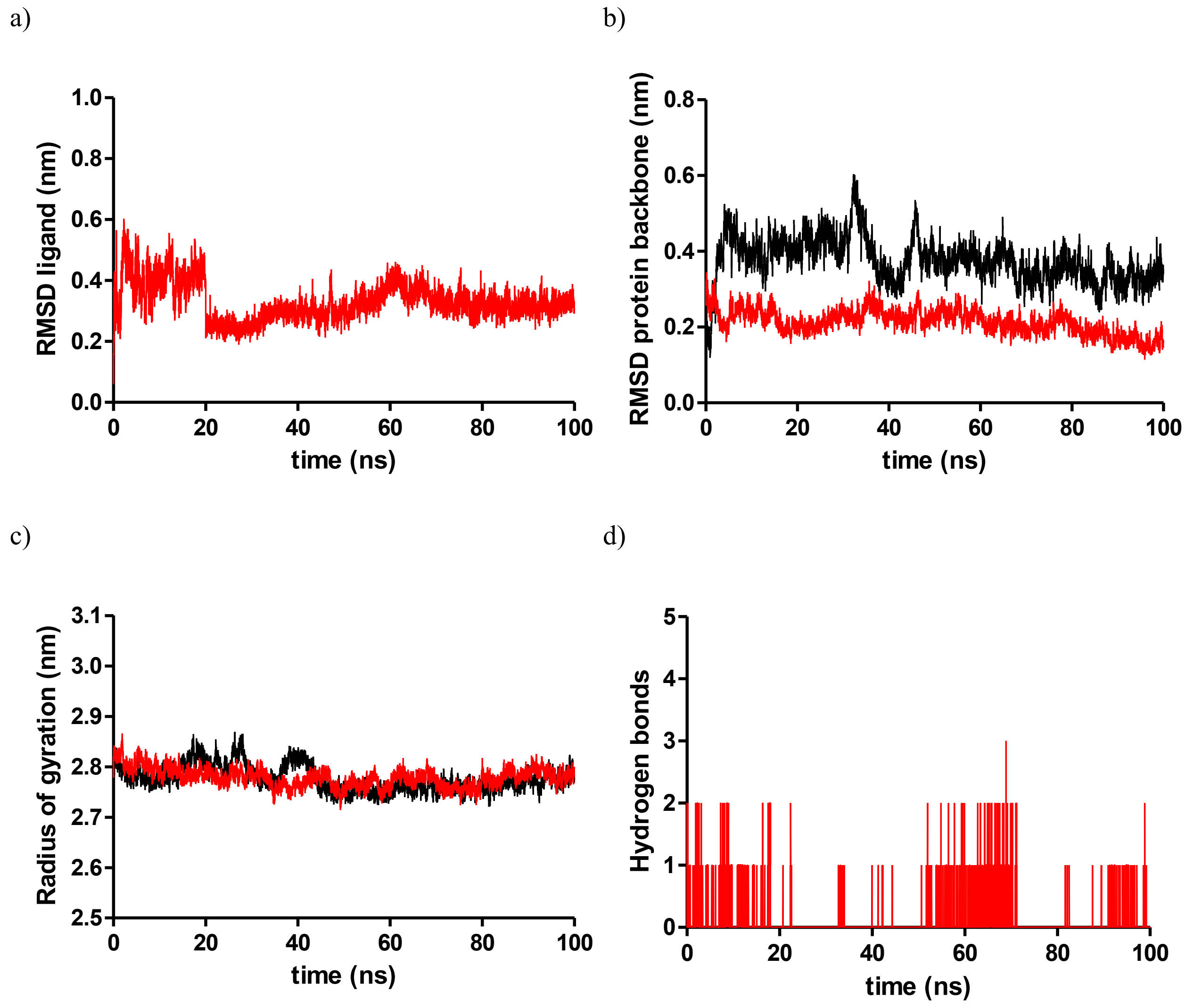

Figure S19. Analysis of the evolution of the complex of **3b[S]** docked in site III of HSA: (a) RMSD of heavy atoms of the ligand; (b) RMSD of HSA backbone apo (black) and in complex with the ligand (red); (c) RG of HSA backbone apo (black) and in complex with the ligand (red); (d) hydrogen bonds between the ligand and HAS.

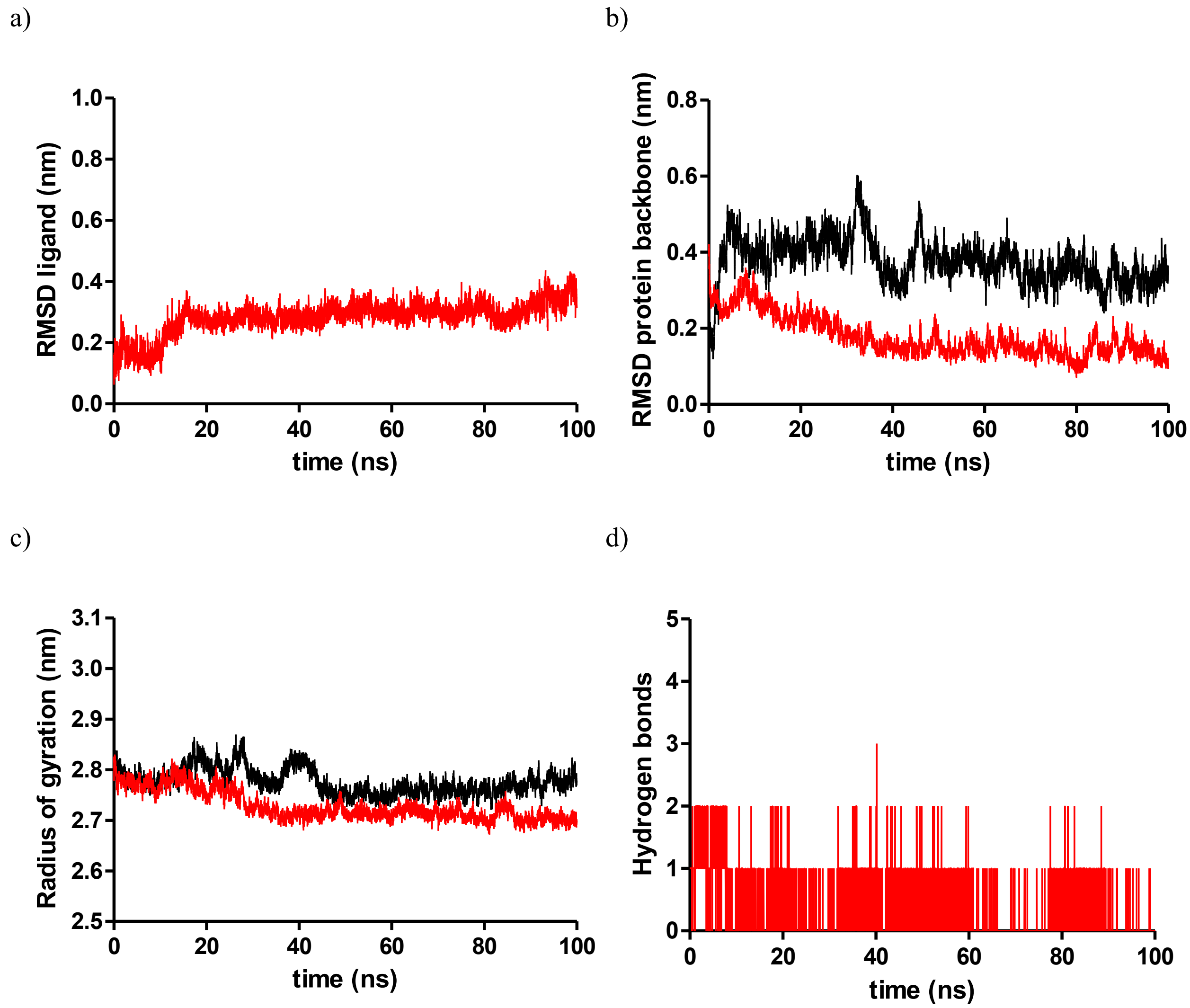

Figure S20. Analysis of the evolution of the complex of **3c[R]** docked in site III of HSA: (a) RMSD of heavy atoms of the ligand; (b) RMSD of HSA backbone apo (black) and in complex with the ligand (red); (c) RG of HSA backbone apo (black) and in complex with the ligand (red); (d) hydrogen bonds between the ligand and HAS.

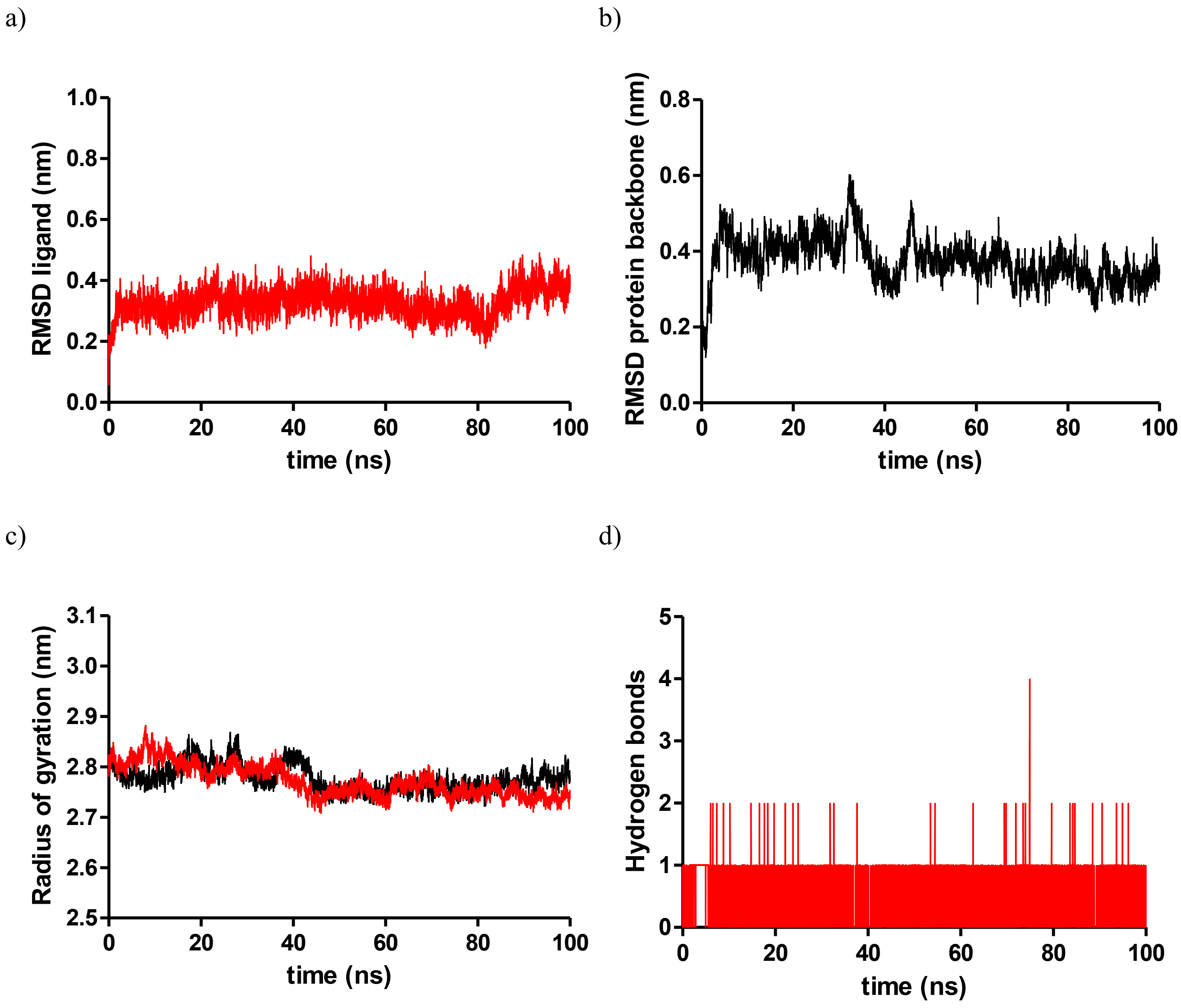

Figure S21. Analysis of the evolution of the complex of **3c[S]** docked in site III of HSA: (a) RMSD of heavy atoms of the ligand; (b) RMSD of HSA backbone apo (black) and in complex with the ligand (red); (c) RG of HSA backbone apo (black) and in complex with the ligand (red); (d) hydrogen bonds between the ligand and HAS.

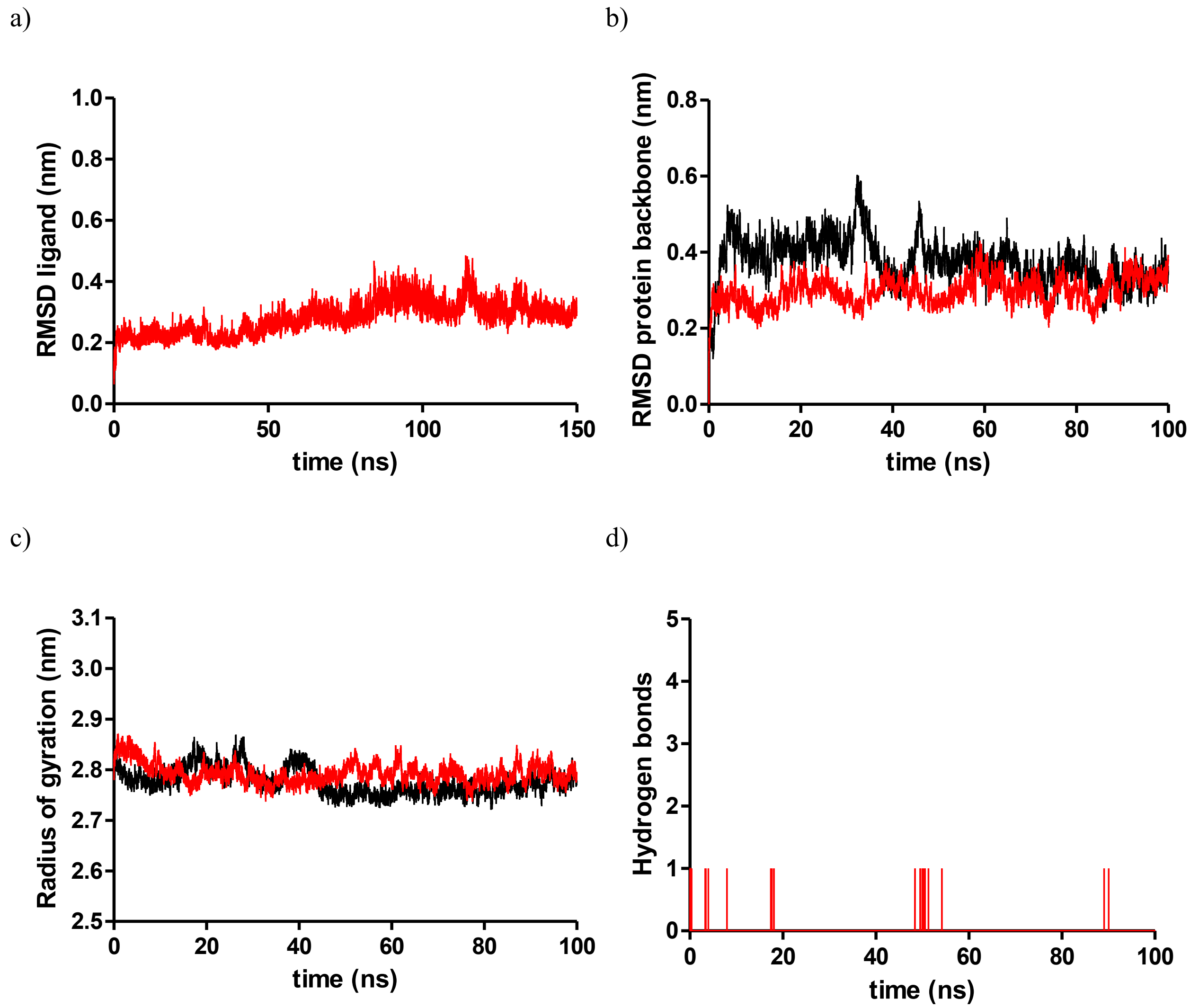

Figure S22. Analysis of the evolution of the complex of **3d[R]** docked in Sudlow site II of HSA: (a) RMSD of heavy atoms of the ligand; (b) RMSD of HSA backbone apo (black) and in complex with the ligand (red); (c) RG of HSA backbone apo (black) and in complex with the ligand (red); (d) hydrogen bonds between the ligand and HAS. Because the RMSD of the heavy atoms of the ligand **3d[R]** (panel a) seems to be increasing in the timeframe between 50 ns and 100 ns of the simulation, the simulation was extended to 150 ns to confirm the stable binding of the ligand **3d[R]** in the pocket Sudlow site II of HSA.

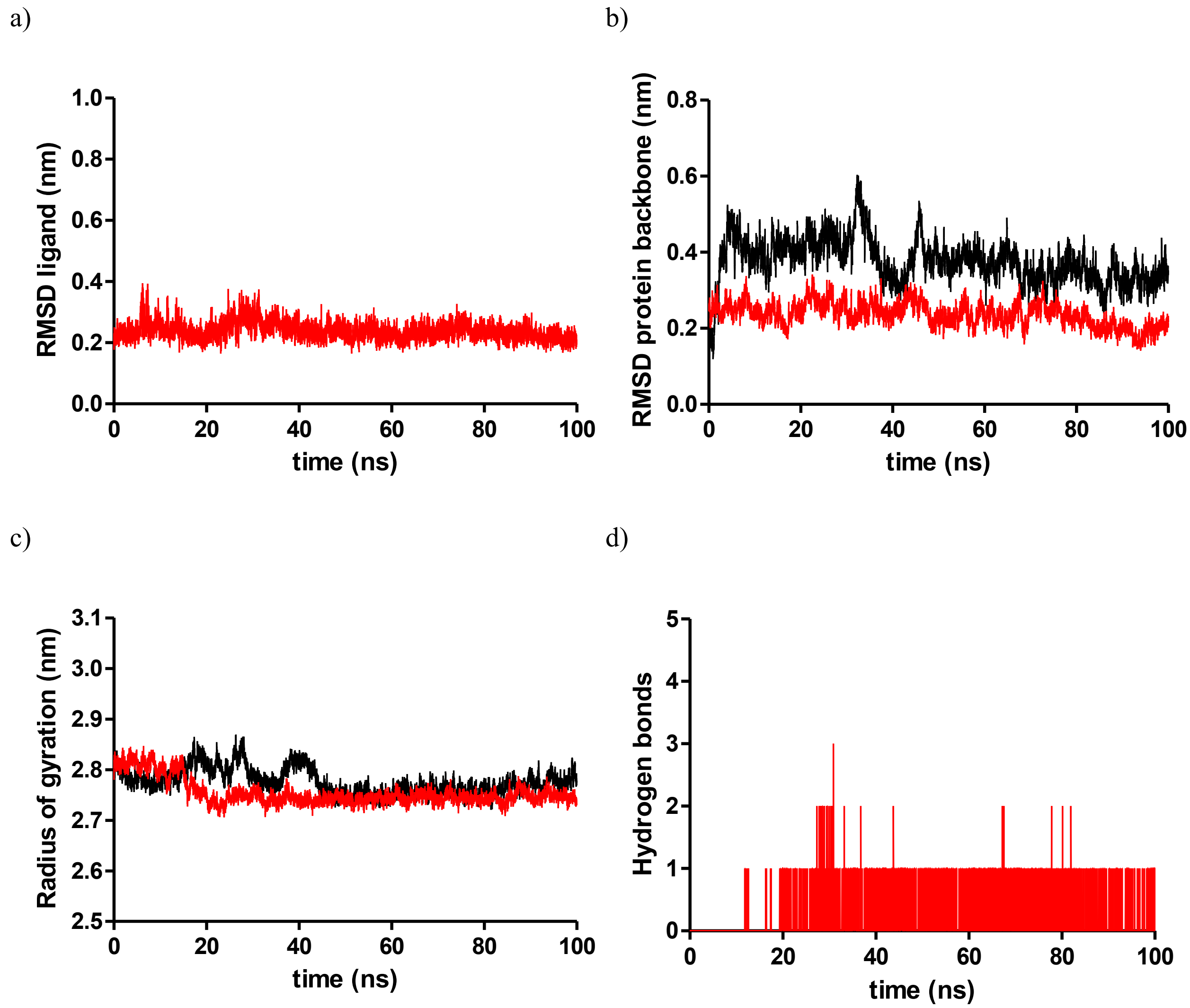

Figure S23. Analysis of the evolution of the complex of **3d[S]** docked in Sudlow site II of HSA: (a) RMSD of heavy atoms of the ligand; (b) RMSD of HSA backbone apo (black) and in complex with the ligand (red); (c) RG of HSA backbone apo (black) and in complex with the ligand (red); (d) hydrogen bonds between the ligand and HAS.

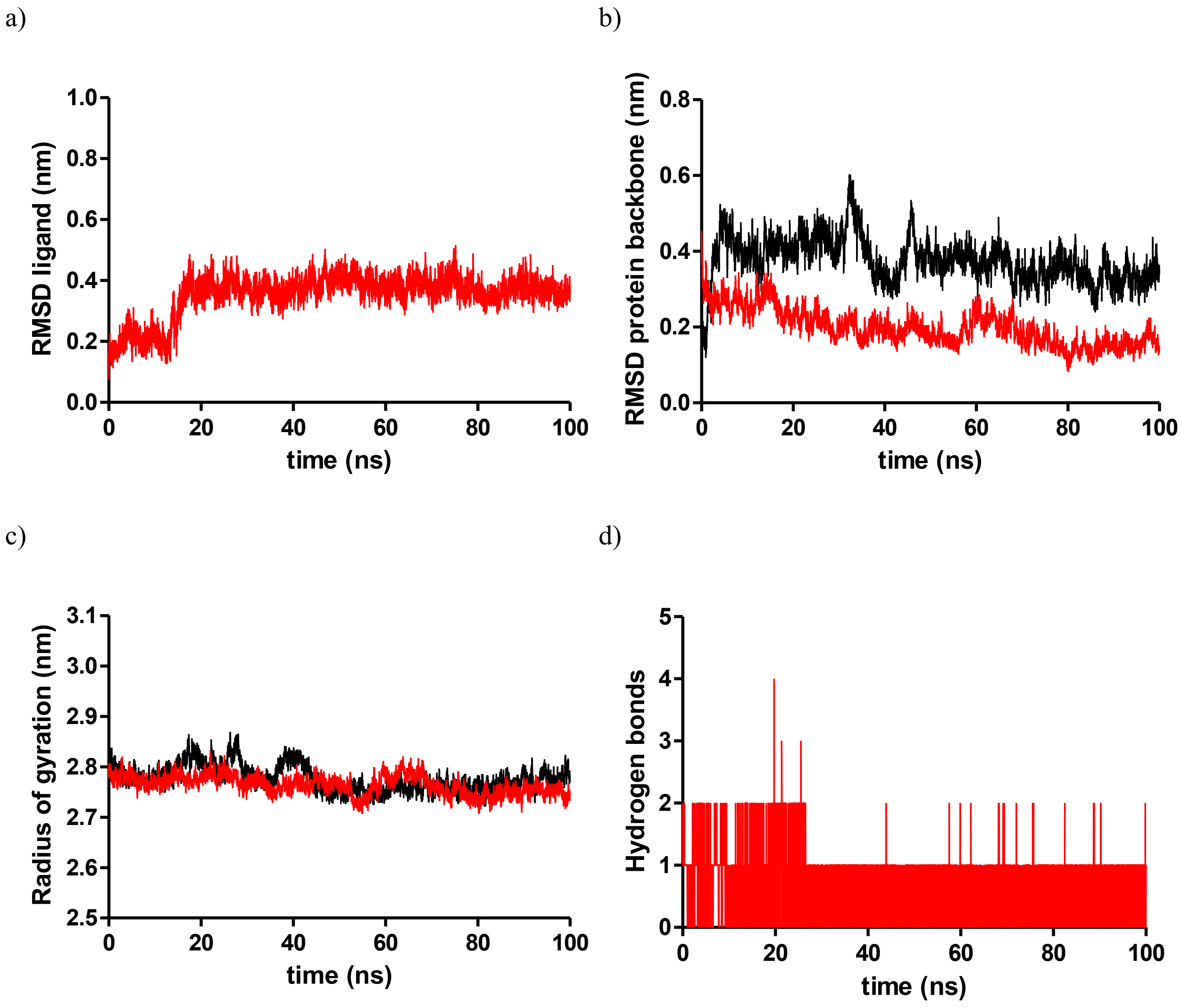

Figure S24. Analysis of the evolution of the complex of **3e[R]** docked in site III of HSA: (a) RMSD of heavy atoms of the ligand; (b) RMSD of HSA backbone apo (black) and in complex with the ligand (red); (c) RG of HSA backbone apo (black) and in complex with the ligand (red); (d) hydrogen bonds between the ligand and HAS.

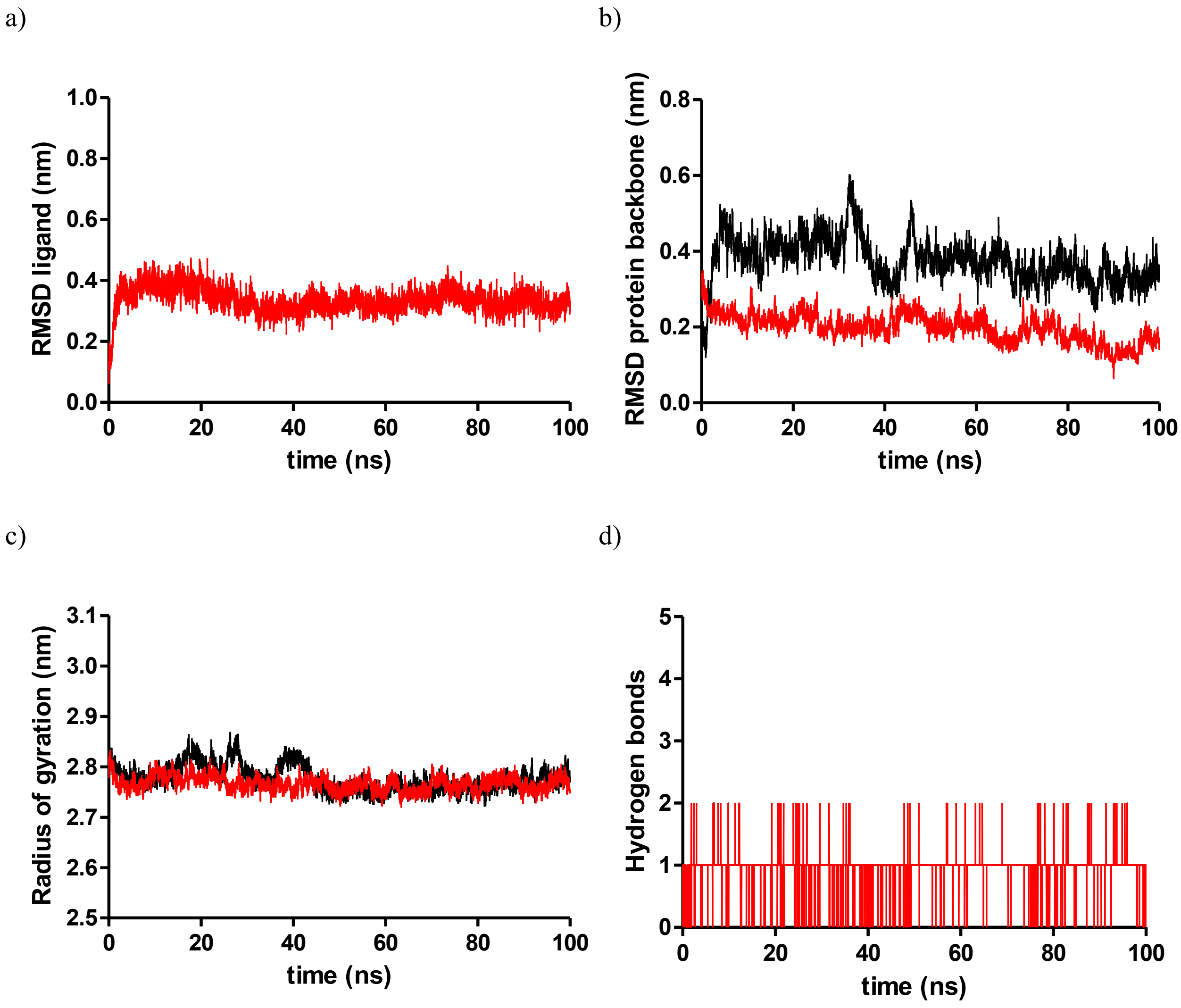

Figure S25. Analysis of the evolution of the complex of **3e[S]** docked in Sudlow site II of HSA: (a) RMSD of heavy atoms of the ligand; (b) RMSD of HSA backbone apo (black) and in complex with the ligand (red); (c) RG of HSA backbone apo (black) and in complex with the ligand (red); (d) hydrogen bonds between the ligand and HAS.

Table S1. Antioxidant activity (HPSA, HRSA) and anti-inflammatory activity, evaluated by inhibition albumin denaturation (IAD) of trimetazidine derivatives (**3a-e**). Results are presented as IC_{50} ± SD/µg/mL. Quercetin (Qrc) and ibuprofen (Ibu) were used as the standards.

Sample	HPSA	HRSA	IAD	R_M ± SD	cLogP	cAnti-I	cAnti-I, ophthalmic
	(IC_{50} ± SD), µg/mL					*Pa*	
**3a**	97.55 ± 1.21	77.98 ± 0.90	110.85 ± 1.17	1.68 ± 0.021	5.09	0.487	0.358
**3b**	93.56 ± 1.29	74.72 ± 0.41	117.41 ± 2.83	1.55 ± 0.015	4.18	0.574	0.414
**3c**	93.07 ± 0.43	71.13 ± 1.33	108.58 ± 2.39	1.61 ± 0.020	4.23	0.482	0.402
**3d**	135.25 ± 13.19	74.59 ± 1.02	115.88 ± 1.54	1.45 ± 0.008	5.16	0.531	0.428
**3e**	99.75 ± 0.75	92.56 ± 5.92	117.51 ± 2.70	1.55 ± 0.016	5.39	0.375	0.362
**Standards**							
**Qrc**	68.07 ± 1.02	70.11 ± 1.17	-	-	-	-	-
**Ibu**	-	-	76.05 ± 1.04	1.11 ± 0.01	3.72	0.901	0.537
